# A Novel Combination Therapy Using Rosuvastatin and *Lactobacillus* Combats Dextran Sodium Sulfate-Induced Colitis in High-Fat Diet-Fed Rats by Targeting the TXNIP/NLRP3 Interaction and Influencing Gut Microbiome Composition

**DOI:** 10.3390/ph14040341

**Published:** 2021-04-08

**Authors:** Sameh Saber, Eslam E. Abd El-Fattah, Galal Yahya, Naglaa A. Gobba, Abdalkareem Omar Maghmomeh, Ahmed E. Khodir, Ahmed A. E. Mourad, Ahmed S. Saad, Hager G. Mohammed, Nehal A. Nouh, Ahmed Shata, Noha A. Amin, Magdy Abou El-Rous, Samuel Girgis, Eman El-Ahwany, Eman M. Khalaf, Attalla F. El-Kott, Ahmed M. El-Baz

**Affiliations:** 1 Department of Pharmacology, Faculty of Pharmacy, Delta University for Science and Technology, Gamasa 11152, Egypt; 2 Department of Biochemistry, Faculty of Pharmacy, Delta University for Science and Technology, Gamasa 11152, Egypt; eslam_620@yahoo.com; 3 Department of Microbiology and Immunology, Faculty of Pharmacy, Zagazig University, Al Sharqia 44519, Egypt; galalmetwally2020@gmail.com; 4 Department of Pharmacology and Toxicology, College of Pharmacy, Misr University for Science and Technology, Giza 12411, Egypt; naglaa.gobba@must.edu.eg or; 5 Department of Biochemistry, Faculty of Pharmacy, Arab Private University for Science and Technology, Hama 1293400, Syria; kareem8933@hotmail.com or; 6 Department of Pharmacology, Faculty of Pharmacy, Horus University-Egypt, New Damietta 34518, Egypt; dr.ahmed_esam@yahoo.com or; 7 Department of Pharmacology and Toxicology, Faculty of Pharmacy, Port-Said University, Port-Said 42511, Egypt; ahmed.mourad@yahoo.com (A.A.E.M.); mosa1200@yahoo.com (A.S.S.); 8 General Administration of Pharmacy, Mansoura 11001, Egypt; hagergamil88@yahoo.com; 9 Department of Microbiology, Albatterjee Medical College, Jeddah 6231, Saudi Arabia; nehalahmed_nouh@yahoo.co.uk; 10 Department of Clinical Pharmacology, Faculty of Medicine, Mansoura University, Mansoura 35516, Egypt; ahmedshata20099@gmail.com; 11 Department of Clinical Pharmacy, Faculty of Pharmacy, Delta University for Science and Technology, Gamasa 11152, Egypt; 12 Department of Haematology, Theodor Bilharz Research Institute, Giza 12411, Egypt; esmaealn78@gmail.com or; 13 Department of Biochemistry, Faculty of Pharmacy, Helwan University, Cairo 11795, Egypt; magdygamal332@yahoo.com or; 14 Department of Pharmaceutics, Faculty of Pharmacy, Alsalam University, Kafr El-Zayat 31612, Egypt; Girgissamuel2@gmail.com; 15 Department of Immunology, Theodor Bilharz Research Institute, Giza 12411, Egypt; ahwany@aucegypt.edu; 16 Department of Microbiology and Immunology, Faculty of Pharmacy, Damanhour University, Damanhour 22511, Egypt; eimanpharmacist@gmail.com; 17 Department of Biology, College of Science, King Khalid University, Abha 61421, Saudi Arabia; elkottaf@yahoo.com; 18 Department of Zoology, Faculty of Science, Damanhour University, Damanhour 22511, Egypt; 19 Department of Microbiology and Biotechnology, Faculty of Pharmacy, Delta University for Science and Technology, Gamasa 11152, Egypt

**Keywords:** ulcerative colitis, NLRP3 inflammasome, rosuvastatin, *Lactobacillus*, gut microbiome, TXNIP, Ox-LDL

## Abstract

Inflammasome targeting and controlling dysbiosis are promising therapeutic approaches to control ulcerative colitis. This report is the first to investigate the mechanisms underlying the coloprotective effects of rosuvastatin and *Lactobacillus* and their combined therapy on dextran sodium sulfate (DSS)-induced colitis in high-fat diet (HFD)-fed rats. Our results demonstrate the aggravation of intestinal inflammation as a consequence of an HFD following DSS administration. An association between dyslipidemia, LDL oxidation, CD36 expression, ROS generation, thioredoxin-interacting protein (TXNIP) upregulation, and NLRP3 inflammasome activation was demonstrated by DSS exposure in HFD-fed rats. We demonstrated that rosuvastatin/*Lactobacillus* significantly suppressed the DSS/HFD-induced increase in colon weight/length ratio, DAI, MDI, and myeloperoxidase, as well as corrected dysbiosis and improved histological characteristics. Additionally, caspase-1 activity and IL-1β-driven pyroptotic activity was significantly reduced. Rosuvastatin/*Lactobacillus* showed prominent anti-inflammatory effects as revealed by the IL-10/IL-12 ratio and the levels of TNF-α and IL-6. These latter effects may be attributed to the inhibition of phosphorylation-induced activation of NF-κB and a concomitant reduction in the expression of NLRP3, pro-IL-1β, and pro-IL-18. Furthermore, rosuvastatin/*Lactobacillus* reduced Ox-LDL-induced TXNIP and attenuated the inflammatory response by inhibiting NLRP3 inflammasome assembly. To conclude, rosuvastatin/Lactobacillus offers a safe and effective strategy for the management of ulcerative colitis.

## 1. Introduction

Inflammatory bowel disease (IBD) represents a global health concern because of the continuous increase in the number of cases. IBDs may be categorized as Crohn’s disease (CD) and ulcerative colitis (UC) [1,2]. UC is a chronic inflammatory disorder defined by colon mucosal inflammation with a peak age of onset in the third decade of life for both men and women [3]. One theory explaining the pathogenesis of IBDs, especially UC, is an imbalance that exists in the gut microbiota, which leads to a persistent inflammatory response [4]. As a component of innate immunity, the inflammasome is a multiprotein oligomer responsible for activating inflammatory reactions, such as those present in UC [5]. Among the various inflammasome subtypes, the nucleotide-binding oligomerization domain (NOD)-, leucine-rich repeat (LRR)- and pyrin domain-containing protein 3 (NLRP3) represents a sensitive sensor of microbial and other danger signals and plays a crucial role in the mucosal immune response to promote the maturation of the pro-inflammatory cytokines, interleukin-1β (IL-1β) and IL-18 [6].

Several putative risk factors have been evaluated for IBD. A high-fat diet (HFD) was discovered as one of the most important risk factors [7]. High-fat diet promotes increases in plasma LDL and Ox-LDL levels [8]. It is worth noting that the effects of increased levels of Ox-LDL on an injured colon have not yet been studied. Additionally, Ox-LDL/CD36/ ROS generation/TXNIP upregulation/NLRP3 inflammasome activation axis warrants investigation during the course of colitis. Moreover, consuming an HFD results in a leaky gut with increased permeability and changes in the microbiota composition [4]. Gut microbiota exist in the trillions in the human gut and play an essential role in protecting the intestinal barrier and maintaining homeostasis of the immune system [9]. Gut bacteria have a symbiotic relationship with the host by providing some fundamental molecules including short-chain fatty acids, vitamins, and conjugated bile acids [10]. Dietary variables may be associated with the course of UC pathogenesis or disease through direct effects on the host or indirect effects by attenuating gut microbiota composition or function. Hence, the diet has a major role in shaping the gut microbial composition [11]. Therefore, aggravation of colon inflammation would be expected in colitis patients consuming high fat diet and suffering from increased plasma levels of Ox-LDL.

Bacterial products bind to pattern recognition receptors such as the Toll-like receptors (TLRs) [12]. Consequently, nuclear transcription factor kappa B (NF-κB) is activated, which stimulates the production of inflammatory cytokines including tumor necrosis factor (TNF-α) and IL-1β. This results in the activation of the inflammatory response, which prevents infection [13,14,15].

A strong association exists between the composition of the intestinal microbiota and the incidence of UC. Factors include decreased biodiversity and altered composition of the fecal and intestinal microbiota of UC patients [16]. Probiotics represent immune-modulating commensal microorganisms that can boost host immunity specifically when administered in the proper quantity. Lactic acid bacteria were shown to provide immune-modulatory functions by altering the gut microbiota [17]. The clinical benefits of using probiotics for the treatment of UC patients has proven significant in the control of UC [18]. *Lactobacillus* (LB) spp. is considered to be one of the most promising probiotics used for the treatment of several diseases including IBDs. They modulate the ecosystem and population of the gut microbiota [17].

Rosuvastatin (RSVT) is a 3-hydroxy-3-methylglutaryl coenzyme A reductase inhibitor. The anti-inflammatory effects of RSVT was demonstrated in the DSS-induced colitis model in which authors stated that regulation of oxidative stress is the underlying mechanism [19]. In addition, Chen, et al. [20] reported that *Lactobacillus fermentum* HY01 showed promising preventive effects on IκBα degradation and inhibited of NF-κB p65 phosphorylation cascades in a mouse model of DSS-induced colitis. However, it has not yet been determined the mechanism through which RSVT or LB affects the development of intestinal ulcerations and microbiome composition in colitic rats fed with an HFD. It should be noted that, an HFD triggers a significant increase in IL-6 levels with a concomitant activation of NF-κB, which is a powerful inflammasome activator [21]. Thus, we investigated the mechanisms underlying the coloprotective effect of RSVT and *Lactobacillus* as monotherapies and in combination in high fat diet-fed rats intoxicated with dextran sodium sulfate. We also were interested in evaluating their effects on the immunomodulation and microbiome composition.

## 2. Results

### 2.1. Effect of RSVT and LB on the Colon Weight/length Ratio, MDI, DAI, and Colonic Myeloperoxidase Activity

**Figure 1:**
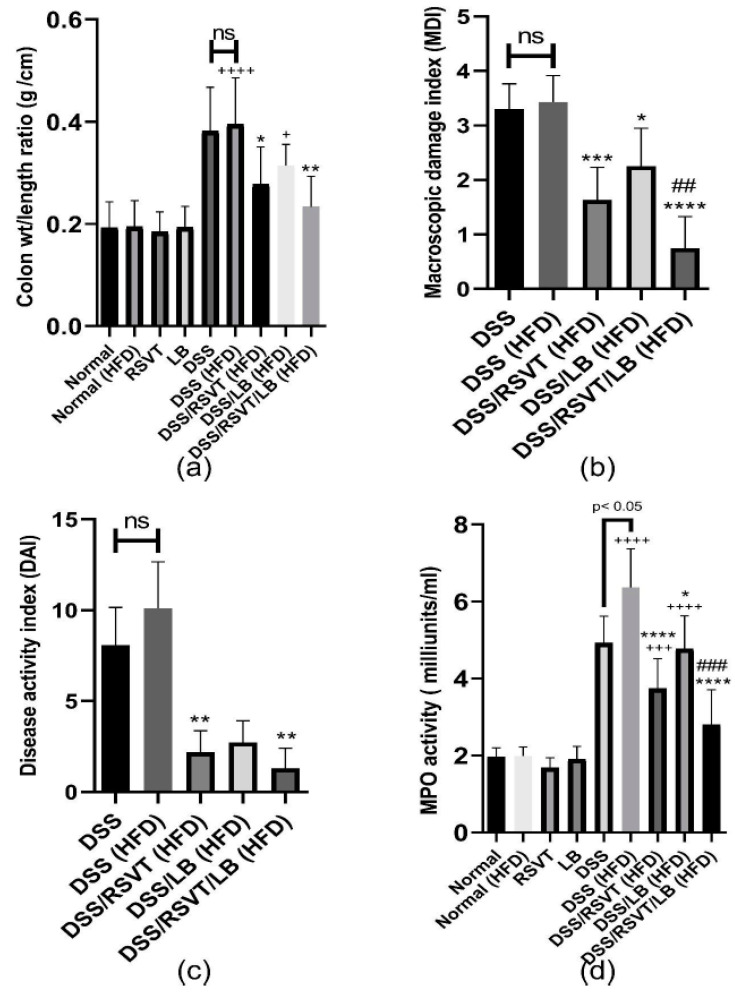
Effect of rosuvastatin (RSVT), *Lactobacillus* (LB), and their combined therapy on (**a**), colon weight/length ratio; (**b**), macroscopic damage index (MDI); (**c**), disease activity index (DAI); (**d**), myeloperoxidase (MPO) activity. +, *p* < 0.05 vs Normal (HFD); +++, *p* < 0.001 vs. Normal (HFD); ++++, *p* < 0.0001 vs. Normal (HFD); *, *p < 0.05* vs DSS (HFD); **, *p <* 0.01 vs. DSS (HFD); ***, *p* < 0.001 vs. DSS (HFD); ****, *p* < 0.0001 vs DSS (HFD); ##, *p* < 0.01 vs. DSS/LB (HFD); ###, *p* < 0.001 vs. DSS/LB (HFD); ns, non-significant.

Concerning the colonic weight/length ratio, the DSS (HFD) group exhibited a significant increase in colonic weight/length ratio compared with the normal (HFD) group (*p* < 0.0001) (Figure 1a). The DSS/RSVT (HFD) and DSS/RSVT/LB (HFD) groups showed a significant offset in the increase of colon weight/length ratio induced by DSS/HFD (*p* < 0.05). However, the colonic weight/length ratio did not differ significantly in the DSS/LB (HFD) rat group compared with that in the DSS/HFD group. As shown in Figure 1b, the MDI in the DSS/HFD group was not significantly different compared with that in the DSS group. The DSS/RSVT (HFD), DSS/LB (HFD), and DSS/RSVT/LB (HFD) groups exhibited a significant decrease in MDI compared with that in the DSS/HFD group (*p* < 0.05). The DSS/RSVT/LB (HFD) group showed a significant decrease in MDI compared with that in the DSS/LB (HFD) group (*p* < 0.05). A combinatorial DAI was used to evaluate disease activity and the response to various treatments. Figure 1c presents DAI resulting from acute colitis induction. The DSS/RSVT (HFD) and DSS/RSVT/LB (HFD) groups showed a significant decrease in the severity of colitis (*p* < 0.01) compared with the DSS/HFD group. However, DAI did not differ significantly in the DSS/LB (HFD) rats compared with that in the DSS/HFD group. As shown in Figure 1d, the DSS/RSVT (HFD) exhibited a significant increase in colon myeloperoxidase (MPO) activity reaching 3.3-fold relative to the normal (HFD) value (*p* < 0.0001). However, MPO activity was significantly reduced in the DSS/RSVT (HFD), DSS/LB (HFD), and DSS/RSVT/LB (HFD) groups by 41.2% (*p* < 0.0001), 28.3% (*p* < 0.05), and 56% (*p* < 0.05), respectively, compared with that in the DSS/HFD group. However, the DSS/RSVT/LB (HFD) group showed a significant decrease in MPO activity compared with that in the DSS/LB (HFD) group (*p* < 0.05). Additionally, DSS (HFD) rats showed a significant increase in MPO activity compared with that in the DSS rats.

### 2.2. Histopathological Examination

**Figure 2:**
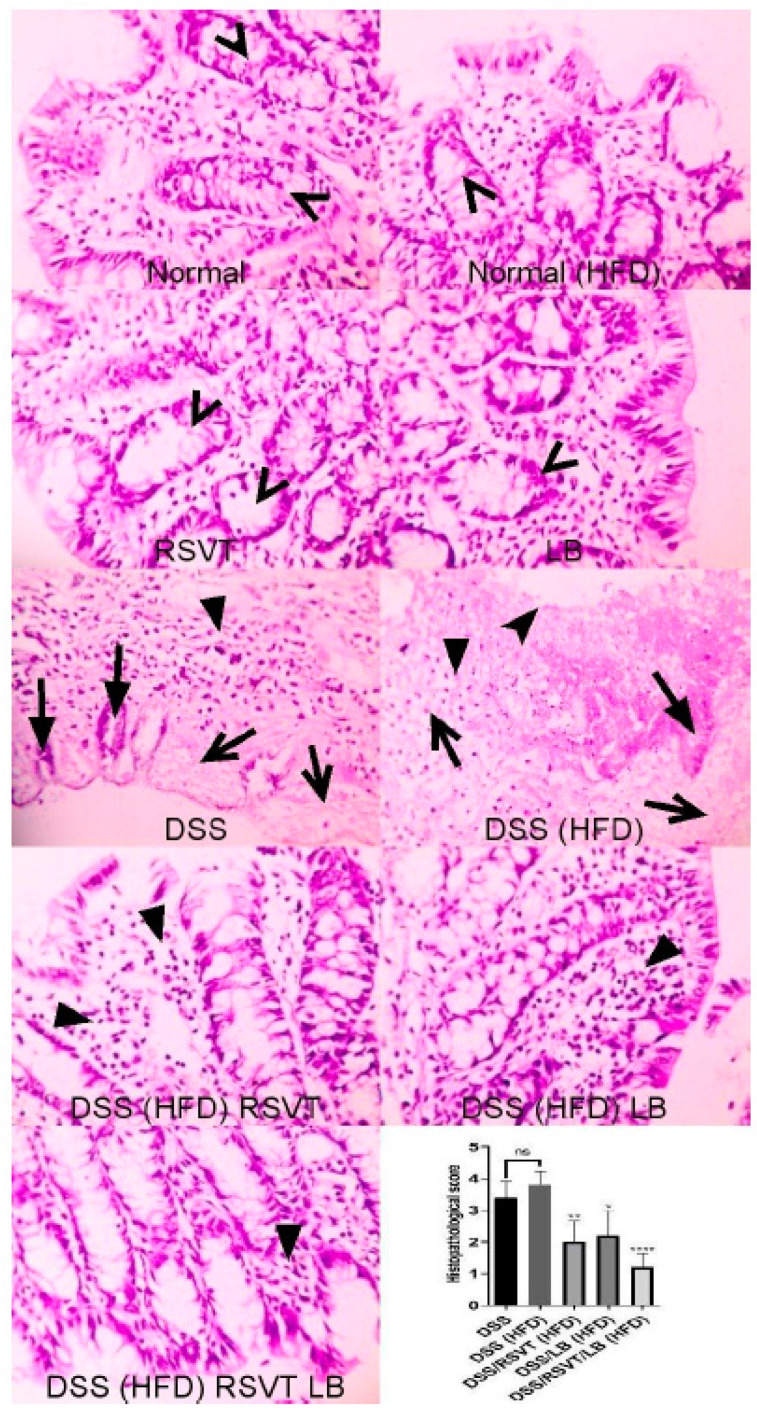
Photomicrographs of colon specimens from Normal, Normal (HFD), RSVT, and LB rat groups displaying normal colonic mucosa and crypts (open arrowheads). Colon specimens from DSS and DSS (HFD) rat groups display de-epithelialization (notched arrowhead), loss of crypts (filled arrows), degenerated tissue (open arrows) and inflammatory cell infiltration (filled arrowhead). Colon specimens from DSS/RSVT (HFD) and DSS/LB (HFD) display moderate crypt degeneration and inflammatory cell infiltration (filled arrowhead). Colon specimens from DSS/RSVT/LB (HFD) display marked decrease in crypt degeneration and inflammatory cell infiltration (filled arrowhead). In addition, DSS/RSVT (HFD), DSS/LB (HFD) and DSS/RSVT/LB (HFD) groups of rats showed a significant decrease in the microscopic evaluation score (*p* < 0.01, *p* < 0.05, *p* < 0.0001, respectively) compared with DSS (HFD) rats. H&E, ×400. *, *p* < 0.05 vs. DSS (HFD); **, *p* < 0.01 vs. DSS (HFD); ****, *p* < 0.0001 vs DSS (HFD); ns, non-significant.

As shown in Figure 2, colon specimens from the Normal, Normal (HFD), RSVT, and LB groups displayed a normal colonic mucosa and crypts. Colon specimens from the DSS and DSS (HFD) rat groups display de-epithelialization, loss of crypts, and inflammatory cell infiltration. Colon specimens from the DSS/RSVT (HFD) and DSS/LB (HFD) groups exhibited moderate crypt degeneration and inflammatory cell infiltration. Colon specimens from the DSS/RSVT/LB (HFD) group displayed a significant decrease in crypt degeneration and inflammatory cell infiltration. Additionally, the DSS/RSVT (HFD), DSS/LB (HFD), and DSS/RSVT/LB (HFD) groups showed a significant decrease in microscopic evaluation score (*p* < 0.01, *p* < 0.05, *p* < 0.0001, respectively) compared with the rats in the DSS (HFD) group.

### 2.3. Effect of RSVT and LB on NF-ĸB p65 and Caspase-1 Immunolabeling

**Figure 3:**
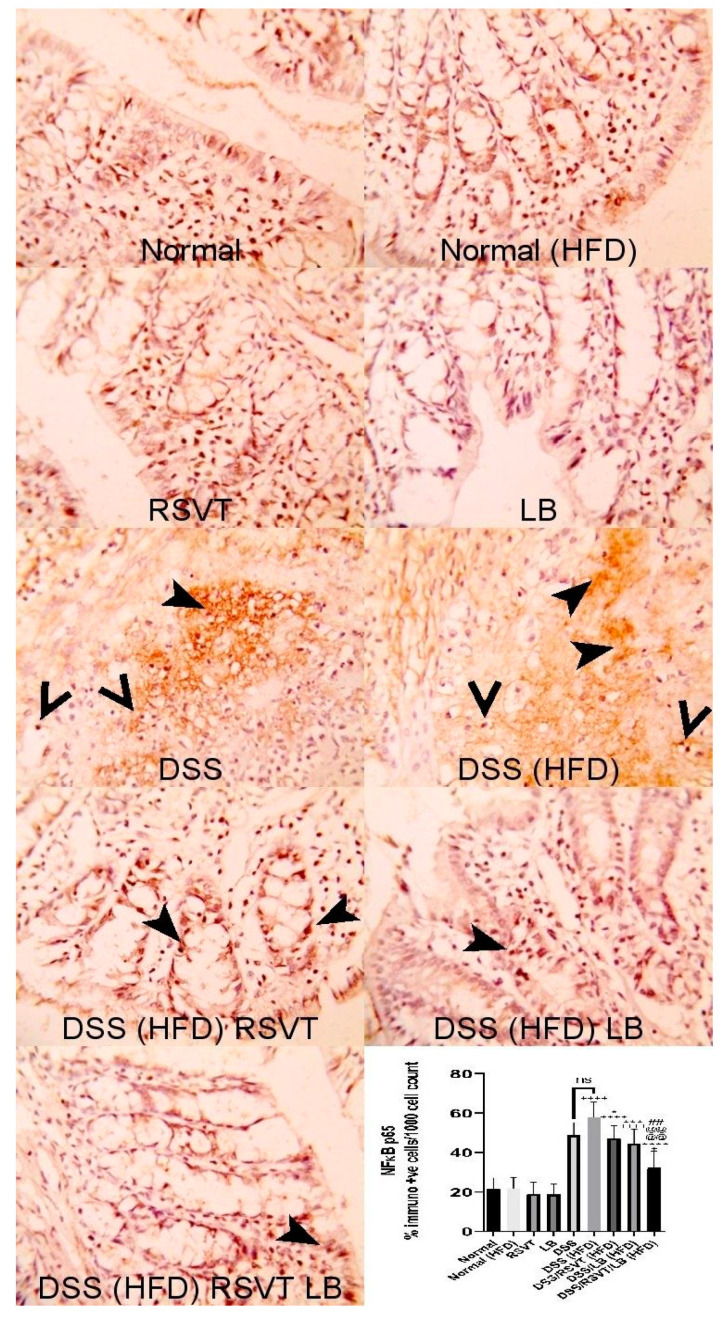
Photomicrographs of colon specimens showing NF-ĸB p65 immunoexpression. Normal, Normal (HFD), RSVT, and LB groups display normal NF-ĸB p65 immunoexpression. The DSS and DSS (HFD) groups display increased immunoexpression (notched arrowheads) and nuclear staining (open arrowheads). DSS/RSVT (HFD), DSS/LB (HFD) and DSS/RSVT/LB (HFD) rat groups showing a significant decrease in NFĸB p65 immunoexpressiom compared with DSS/HFD (*p* < 0.05, *p* < 0.001, and *p* < 0.0001, respectively). IHC, ×400. +, *p* < 0.05 vs. Normal (HFD); ++++, *p* < 0.0001 vs. Normal (HFD); *, *p* < 0.05 vs. DSS (HFD); ***, *p* < 0.001 vs. DSS (HFD); ****, *p* < 0.0001 vs. DSS (HFD); ##, *p* < 0.01 vs. DSS/LB (HFD); @@@@, *p* < 0.0001 vs. DSS/RSVT (HFD); ns, non-significant.

**Figure 4:**
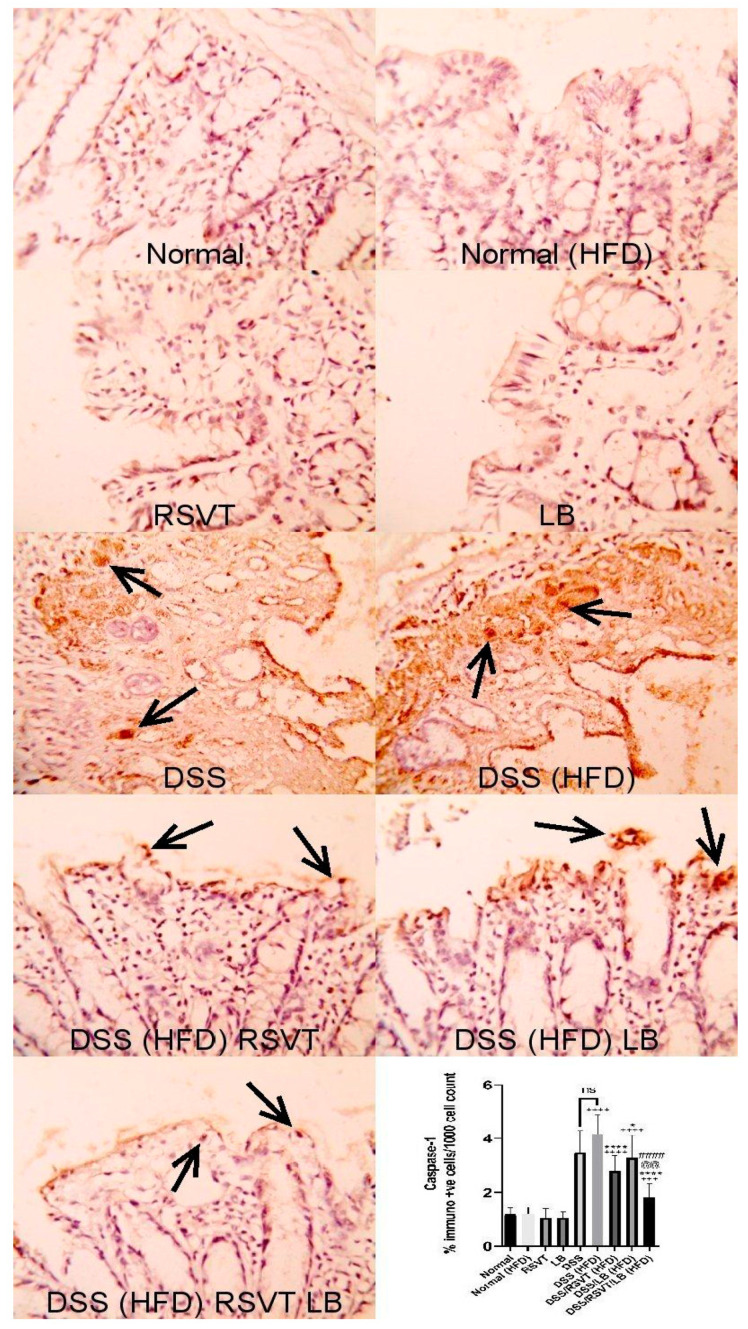
Photomicrographs of colon specimens showing caspase-1 immunoexpression. Normal, Normal (HFD), RSVT, and LB groups display normal caspase-1 immunoexpression. The DSS and DSS (HFD) groups display increased immunoexpression (open arrows). DSS/RSVT (HFD), DSS/LB (HFD) and DSS/RSVT/LB (HFD) rat groups showing a significant decrease in caspase-1immunoexpressiom compared with DSS/HFD. IHC, ×400. +++, *p* < 0.001 vs. Normal (HFD); ++++, *p* < 0.0001 vs. Normal (HFD); *, *p* < 0.05 vs. DSS (HFD); ****, *p* < 0.0001 vs. DSS (HFD); ####, *p* < 0.0001 vs. DSS/LB (HFD); @@, *p* < 0.01 vs. DSS/RSVT (HFD); ns, non-significant.

As shown in Figure 3, NF-ĸB p65 protein expression in the DSS (HFD) group was significantly higher than that in the Normal (HFD) group. The DSS/RSVT (HFD), DSS/LB (HFD), and DSS/RSVT/LB (HFD) groups exhibited a significant decrease in NFĸB p65 expression compared with the DSS/HFD group (*p* < 0.05, *p* < 0.001, and *p* < 0.0001, respectively). As shown in Figure 4, caspase-1 expression in the DSS (HFD) group was significantly higher compared with that in the Normal (HFD) group. The DSS/RSVT (HFD), DSS/LB (HFD), and DSS/RSVT/LB (HFD) rat groups showed a significant decrease in caspase-1 expression compared with the DSS/HFD group (*p* < 0.0001, *p* < 0.05, and *p* < 0.0001, respectively).

### 2.4. Effect of RSVT and LB on Caspase-1 Activity

**Figure 5:**
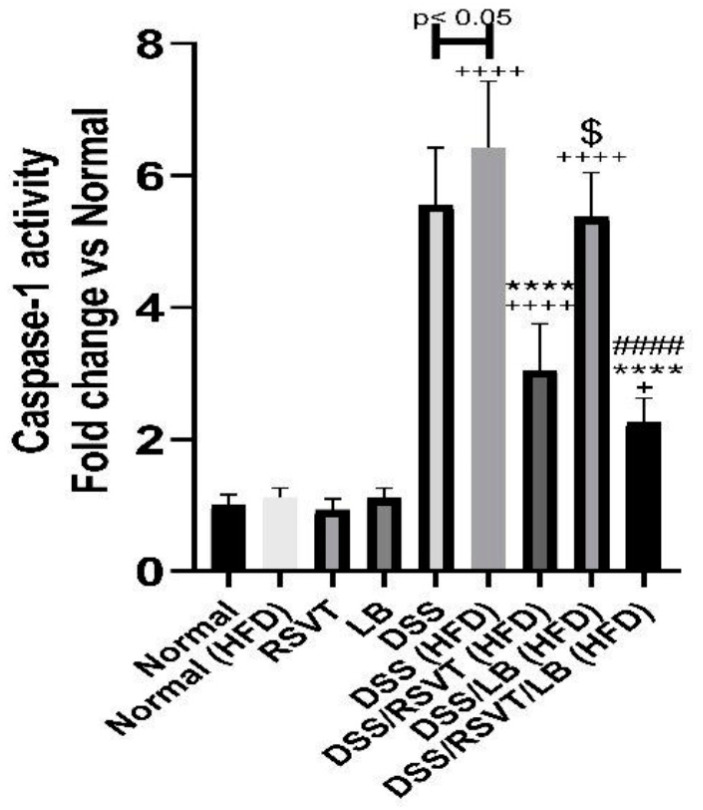
Effect of rosuvastatin (RSVT), *Lactobacillus* (LB), and their combined therapy on caspase-1. +, *p* < 0.05 vs. Normal (HFD); ++++, *p* < 0.0001 vs Normal (HFD); ****, *p* < 0.0001 vs. DSS (HFD); ####, *p* < 0.0001 vs. DSS/LB (HFD); $, *p* < 0.05 DSS/RSVT (HFD) vs. DSS/LB (HFD).

As shown in Figure 5, the DSS (HFD) rat group showed a significant increase in caspase-1 activity compared with the Normal (HFD) (*p* < 0.0001) and DSS (*p* < 0.05) groups. Rats from the DSS/RSVT (HFD) and DSS/RSVT/LB (HFD) groups exhibited a significant counterbalance in increased caspase-1 activity induced by DSS relative to HFD feeding (*p* < 0.0001). However, compared with that in the DSS (HFD) group, caspase-1 activity did not differ significantly in the DSS/LB (HFD) group. The DSS/RSVT (HFD) group exhibited a significant decrease in caspase-1 activity compared with the DSS/LB (HFD) group (*p* < 0.05). Additionally, the DSS/RSVT/LB (HFD) group showed a significant decrease in caspase-1 activity compared with the DSS/LB (HFD) group (*p* < 0.0001).

### 2.5. Effect of RSVT and LB on MDA, SOD, GSH, ROS, and sOx-LDL

**Figure 6:**
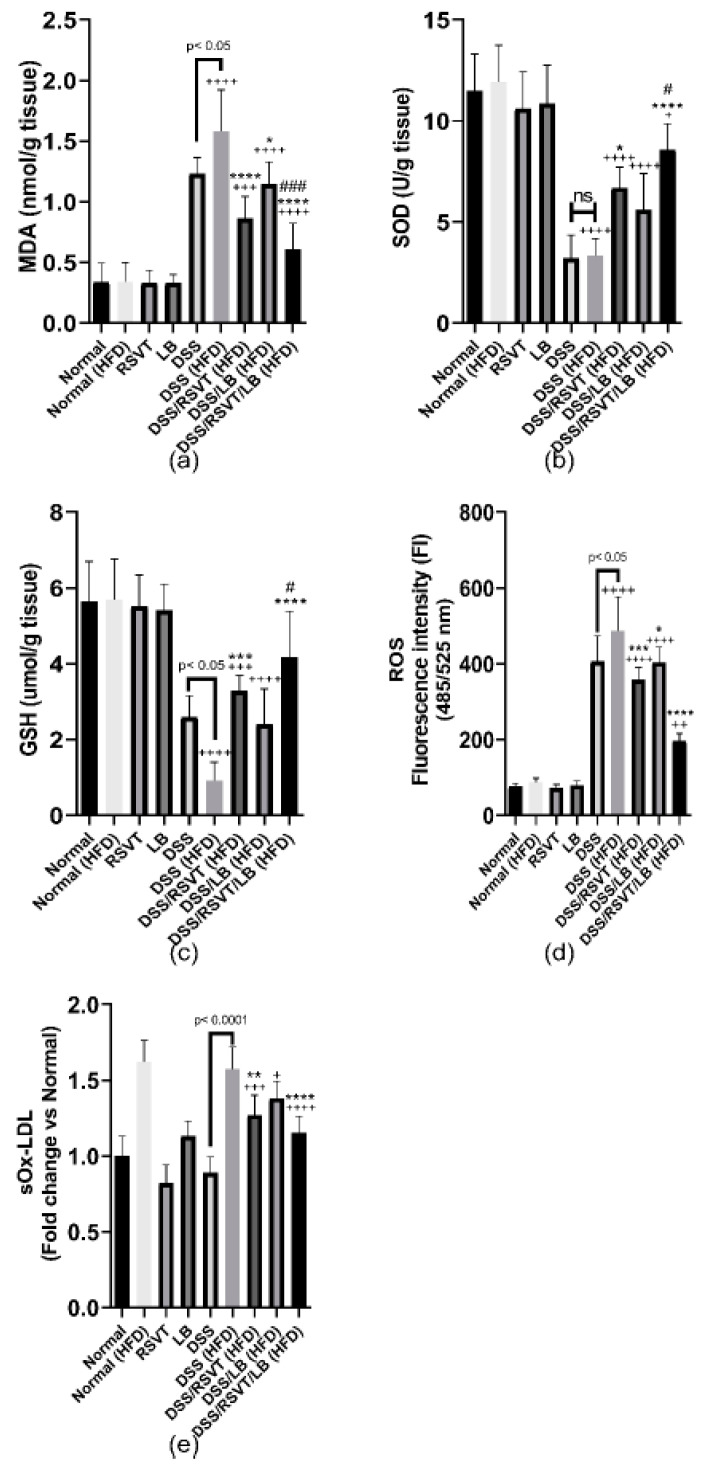
Effect of rosuvastatin (RSVT), *Lactobacillus* (LB), and their combined therapy on (**a**), Malondialdehyde (MDA); (**b**), superoxide dismutase (SOD); (**c**), reduced glutathione (GSH); (**d**), reactive oxygen species (ROS); and (**e**), soluble oxidized low-density lipoprotein (sOx-LDL). +, *p* < 0.05 vs. Normal (HFD); ++, *p* < 0.01 vs. Normal (HFD); +++, *p* < 0.001 vs. Normal (HFD); ++++, *p* < 0.0001 vs. Normal (HFD); *, *p* < 0.05 vs. DSS (HFD); **, *p* < 0.01 vs. DSS (HFD); ***, *p* < 0.001 vs. DSS (HFD); ****, *p* < 0.0001 vs. DSS (HFD); #, *p* < 0.05 vs. DSS/LB (HFD); ###, *p* < 0.001 vs. DSS/LB (HFD); ns, non-significant.

The DSS (HFD) group displayed a significant increase in colonic MDA content, reaching 4.63-fold relative to that of the HFD control group (*p* < 0.0001). Furthermore, compared with that in the DSS (HFD) rat group (*p* < 0.05), the MDA content was significantly decreased in the DSS/RSVT (HFD), DSS/LB (HFD), and DSS/RSVT/LB (HFD) groups. DSS-induced oxidative stress in the rat colons was evaluated by measuring SOD and GSH levels. As shown in Figure 6b,c, the DSS (HFD) group showed a significant reduction in the levels of SOD and GSH by approximately 72.26% and 83.9%, respectively, compared with the Normal (HFD) control group (*p* < 0.0001). Additionally, in comparison with the DSS (HFD) rat group, SOD and GSH levels were significantly increased in the DSS/RSVT (HFD) and DSS/RSVT/LB (HFD) groups. However, the DSS/RSVT/LB (HFD) group exhibited a significant increase in both SOD and GSH than did DSS/LB (HFD) group (*p* < 0.05). SOD and GSH levels did not differ significantly in the DSS/LB (HFD) group than in the DSS (HFD) group. Consistent with the SOD and GSH results, we observed that ROS FI was significantly increased in the DSS (HFD) group than in the Normal (HFD) group. Furthermore, treatment of the DSS (HFD) rats with RSVT, LB, and a combination of both significantly repressed the ROS-induced increase. Moreover, the DSS (HFD) rat group showed a significant increase in ROS compared with that of the DSS group (Figure 6d). Our results also revealed a significant increase in sOx-LDL in the DSS (HFD) group than in the DSS rats and an insignificant change compared with that of the Normal (HFD) group. However, the DSS/RSVT (HFD) and DSS/RSVT/LB (HFD) groups, and not the DSS/LB (HFD) group, exhibited significantly reduced levels of sOx-LDL compared with that of the DSS (HFD) group (Figure 6e).

### 2.6. Effect of RSVT and LB on NF-κB p65, NLRP3, and TXNIP mRNA Expression

**Figure 7:**
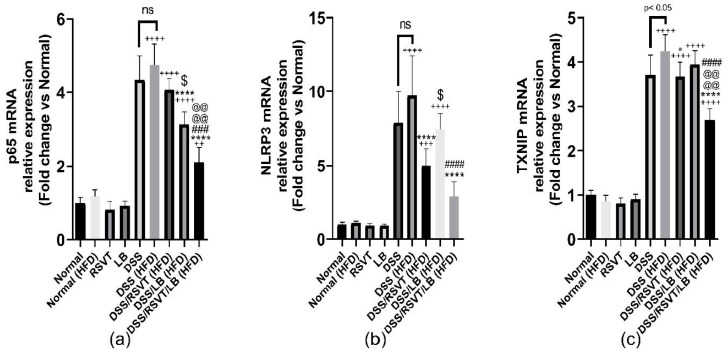
Effect of rosuvastatin (RSVT), *Lactobacillus* (LB), and their combined therapy on (**a**–**c**), the mRNA gene expression of p65, NLRP3, and TXNIP, respectively. ++, *p < 0.01* vs Normal (HFD); +++, *p* < 0.001 vs. Normal (HFD); ++++, *p* < 0.0001 vs. Normal (HFD); *, *p* < 0.05 vs. DSS (HFD); ****, *p <* 0.0001 vs. DSS (HFD); ###, *p* < 0.001 vs. DSS/LB (HFD); ####, *p* < 0.0001 vs. DSS/LB (HFD); @@@@, *p* < 0.0001 vs. DSS/RSVT (HFD); $, *p* < 0.05 DSS/RSVT (HFD) vs. DSS/LB (HFD); ns, non-significant.

DSS (HFD) induced a significant upregulation in the expression of NF-κB p65, NLRP3, and TXNIP mRNA (Figure 7a–c). The DSS/RSVT (HFD) and DSS/RSVT/LB (HFD) rat groups showed a significant decrease in the expression of NLPR3 compared with that of the DSS (HFD) group. However, the DSS/LB (HFD) group did not significantly affect the level of NLPR3 expression. The DSS/RSVT/LB (HFD) group exhibited a significant decrease in NLPR3 mRNA compared with that of the DSS/LB (HFD) group (*p* < 0.05). The DSS/RSVT (HFD) group showed a significant decrease in NLPR3 expression compared with the DSS/LB (HFD) group (*p* < 0.05) (Figure 7a).

By contrast, compared with the DSS (HFD) group, the DSS/LB (HFD) and DSS/RSVT/LB (HFD) groups showed a significant decrease in NF-κB p65 mRNA expression levels. However, the DSS/RSVT (HFD) group did not show a significant change in the level of NF-κB p65 mRNA expression. The DSS/LB (HFD) treatment group exhibited a significant downregulation in NF-κB p65 expression compared with the DSS/RSVT (HFD) treatment group (*p* < 0.05). The DSS/RSVT/LB (HFD) group showed a significant downregulation in NF-κB p65 mRNA expression compared with both the DSS/LB (HFD) and DSS/RSVT (HFD) groups (*p* < 0.05) (Figure 7b). The DSS/RSVT (HFD) and DSS/RSVT/LB (HFD) groups showed a significant decrease in TXNIP mRNA expression compared with the DSS/HFD group. However, compared with the DSS (HFD) group, the DSS/LB (HFD) group did not show a significant change in the level of TXNIP expression. Finally, the DSS/RSVT/LB (HFD) group showed a significant downregulation of TXNIP mRNA gene compared with both the DSS/RSVT (HFD) and DSS/LB (HFD) groups (*p* < 0.05) (Figure 7c).

### 2.7. Effect of RSVT and LB on the Inflammatory Mediators TNF-α, IL-6, IL-10/IL-12 p70 Ratio, NF-κB p-p65/p65 Ratio, IL-1β, IL-18, TXNIP, NLRP3, sCD36, Colon CD36, and Ox-LDL

**Figure 8:**
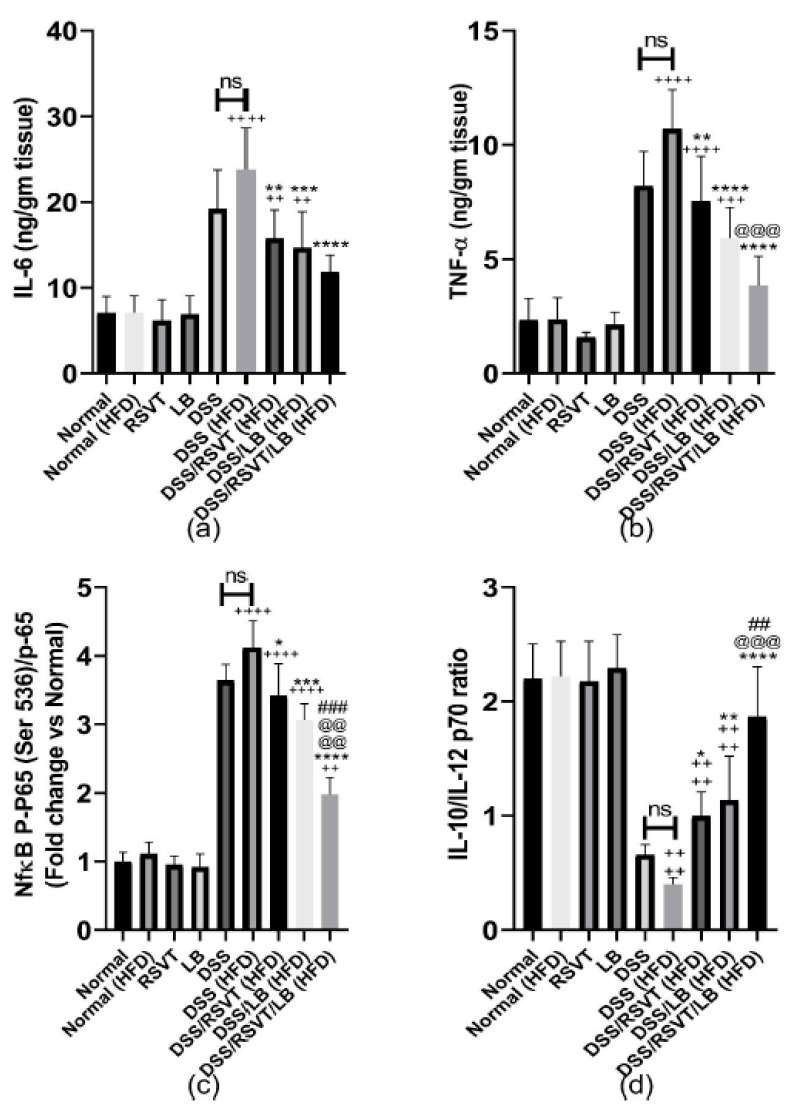
Effect of rosuvastatin (RSVT), *Lactobacillus* (LB), and their combined therapy on (**a**), IL-6; (**b**), tumor necrosis factor alpha (TNF-α); (**c**), NF-κB p-p65 (Ser 536)/p65; and (**d**), IL-10/IL-12 P70. ++, *p* < 0.01 vs. Normal (HFD); +++, *p* < 0.001 vs. Normal (HFD); ++++, *p* < 0.0001 vs. Normal (HFD); *, *p* < 0.05 vs. DSS (HFD); **, *p* < 0.01 vs. DSS (HFD); ***, *p* < 0.001 vs. DSS (HFD); ****, *p* < 0.0001 vs. DSS (HFD); ##, *p* < 0.01 vs. DSS/LB (HFD); ###, *p* < 0.001 vs. DSS/LB (HFD); @@@, *p* < 0.001 vs. DSS/RSVT (HFD); @@@@, *p* < 0.0001 vs. DSS/RSVT (HFD); ns, non-significant.

**Figure 9:**
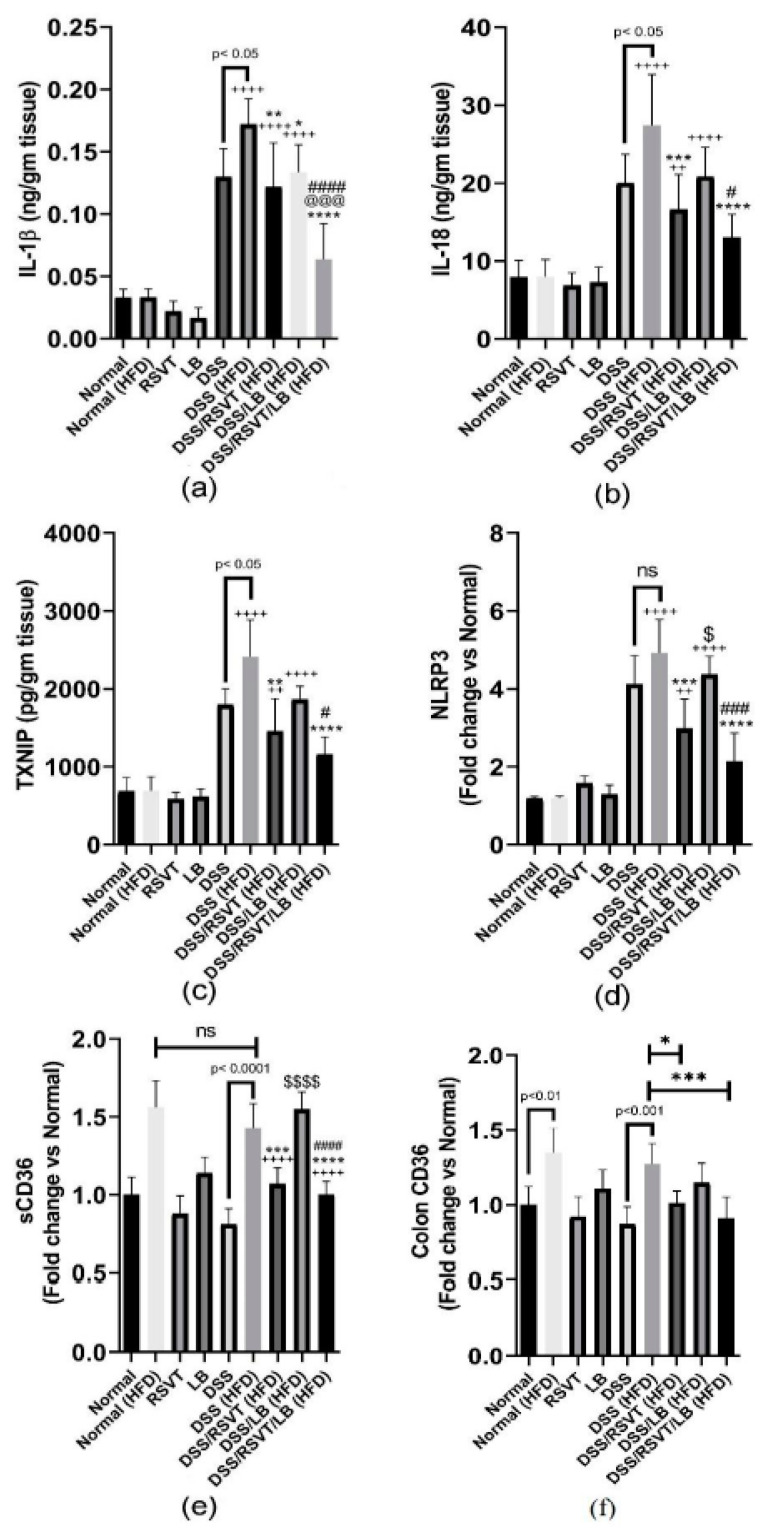
Effect of rosuvastatin (RSVT), *Lactobacillus* (LB), and their combined therapy on (**a**), IL-1β; (**b**), IL-18; (**c**), TXNIP; (**d**), NLRP3; (**e**), sCD36; and (**f**), colon CD36. ++, *p* < 0.01 vs. Normal (HFD); ++++, *p* < 0.0001 vs. Normal (HFD); *, *p* < 0.05 vs. DSS (HFD); **, *p* < 0.01 vs. DSS (HFD); ***, *p* < 0.001 vs. DSS (HFD); ****, *p* < 0.0001 vs. DSS (HFD); #, *p* < 0.05 vs. DSS/LB (HFD); ###, *p* < 0.001 vs. DSS/LB (HFD); ####, *p* < 0.0001 vs. DSS/LB (HFD); @@@, *p* < 0.001 vs. DSS/RSVT (HFD); $, *p* < 0.05 DSS/RSVT (HFD) vs. DSS/LB (HFD); $$$$, *p* < 0.0001 DSS/RSVT (HFD) vs. DSS/LB (HFD); ns, non-significant.

As shown in Figure 8, the DSS (HFD) group induced a significant increase in colon associated inflammatory-related markers (IL-6, Figure 8a; TNF-α, Figure 8b; and NF-κB p-p65/p65 ratio, Figure 8c) compared with the Normal (HFD) control group (*p* < 0.0001). By contrast, the IL-10/IL-12 p70 ratio (Figure 8d) was significantly decreased compared with that of the Normal (HFD) control animals (*p* < 0.0001). The DSS/RSVT (HFD), DSS/LB (HFD), and DSS/RSVT/LB (HFD) groups exhibited a significant decrease in IL-6, TNF-α, and the NF-κB p-p65/p65 ratio, whereas a significant increase in the IL-10/IL-12 p70 ratio was observed compared with the DSS (HFD) group. The DSS/RSVT/LB (HFD) group showed a significant decrease in TNF-α and NF-κB p-p65/p65 ratio, whereas a significant increase in the IL-10/IL-12 p70 ratio was observed compared with that of the DSS/RSVT (HFD) group (*p* < 0.001). The DSS/RSVT/LB (HFD) group exhibited a significant decrease in NF-κB p-p65/p65 ratio, whereas a significant increase in the IL-10/IL-12 p70 ratio was observed compared with that of the DSS/LB (HFD) group (*p* < 0.01). Figure 9 shows a significant increase in colon IL-1β (Figure 9a), IL-18 (Figure 9b), TXNIP (Figure 9c), and NLRP3 (Figure 9d) in the DSS/HFD group compared with that in the Normal (HFD) control group (*p* < 0.0001). By contrast, the IL-10/IL-12 p70 ratio was significantly decreased compared with that in the Normal (HFD control animals (*p* < 0.0001). The DSS/RSVT (HFD), DSS/LB (HFD), or DSS/RSVT/LB (HFD) groups exhibited a significant decrease in the levels of IL-6, TNF-α, and NF-κB p-p65/p65 ratio, whereas a significant increase in the IL-10/IL-12 p70 ratio was observed compared with that in the DSS/HFD group. The DSS/RSVT (HFD) and DSS/RSVT/LB (HFD) groups showed a significant decrease in the levels of IL-1β, IL-18, TXNIP, and NLRP3 compared with the DSS/HFD group (*p* < 0.01), whereas the DSS/LB (HFD) group showed a significant decrease in IL-1β compared with the DSS/HFD group. No significant differences in IL-18, TXNIP, or NLRP3 were observed. The DSS/RSVT/LB (HFD) exhibited a significant decrease in IL-1β, IL-18, TXNIP, and NLRP3 compared with the DSS/LB (HFD) group (*p* < 0.05). Compared with DSS/RSVT (HFD) group (*p* < 0.01), the DSS/RSVT/LB (HFD) showed a significant decrease in IL-1β. Treatment with DSS/RSVT (HFD) resulted in a significant decrease in NLRP3 compared with that of the DSS/LB (HFD) group (*p* < 0.05). Additionally, as shown in Figure 9e, compared with the DSS/RSVT (HFD) and DSS/RSVT/LB (HFD) treatment groups (*p* < 0.001), a significant increase in soluble CD36 (sCD36) was observed in the DSS/HFD group. The DSS/RSVT (HFD) and DSS/RSVT/LB (HFD) groups showed a significant decrease in sCD36 compared with the DSS/LB (HFD) group. Similar to sCD36, the colons of the DSS (HFD) rats showed a significant increase in CD36 compared with those of the DSS rats. Colon CD36 levels were significantly decreased in the DSS/RSVT (HFD) and DSS/RSVT/LB (HFD) groups compared with that in the DSS (HFD) group (Figure 9f).

### 2.8. Effect of RSVT and LB on Lipid Profiles

**Figure 10:**
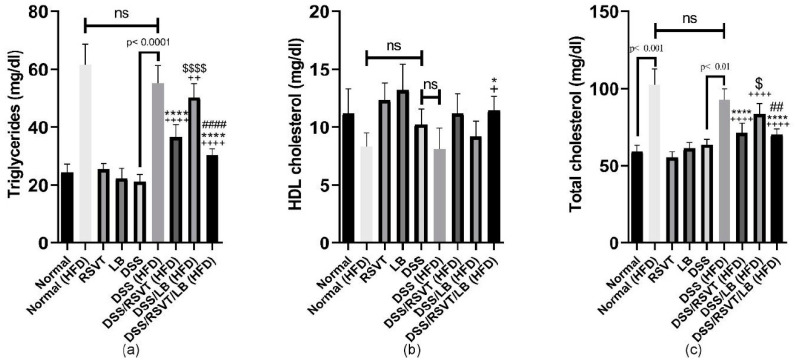
Effect of rosuvastatin (RSVT), *Lactobacillus* (LB), and their combined therapy on (**a**), triglycerides; (**b**), high density lipoprotein (HDL); and (**c**), total cholesterol. +, *p* < 0.05 vs. Normal (HFD); ++, *p* < 0.01 vs. Normal (HFD); ++++, *p* < 0.0001 vs. Normal (HFD); *, *p* < 0.05 vs. DSS (HFD); ****, *p* < 0.0001 vs. DSS (HFD); ##, *p* < 0.01 vs. DSS/LB (HFD); ####, *p* < 0.0001 vs. DSS/LB (HFD); $, *p* < 0.05 DSS/RSVT (HFD) vs. DSS/LB (HFD); $$$$, *p* < 0.0001 DSS/RSVT (HFD) vs. DSS/LB (HFD); ns, non-significant.

The DSS (HFD) rat group exhibited a significant increase in triglyceride (TG) levels (Figure 10a), whereas a significant decrease in HDL (Figure 10b) and no significant change in total cholesterol levels was observed compared with the Normal (HFD) control group (*p* < 0.05) (Figure 10c). Furthermore, total cholesterol and TG levels were significantly decreased in the DSS/RSVT (HFD) and DSS/RSVT/LB (HFD) groups compared with the DSS (HFD) group (*p* < 0.05). Additionally, the DSS/RSVT/LB (HFD) group showed a significant decrease in total cholesterol and TG levels compared with the DSS/LB (HFD) group (*p* < 0.01), whereas the DSS/RSVT (HFD) rats exhibited a significant decrease in total cholesterol and TG levels compared with the DSS/LB (HFD) group (*p* < 0.05).

### 2.9. Effect of RSVT and LB on Microbiome Composition as Determined by Conventional PCR Analysis

Using uniplex PCR, colon tissue samples from the different rat groups were examined for 15 different bacterial species closely linked to UC: *E. faecalis*, *E. faecium*, *E. coli, Bifidobacterium* spp., *P. aeruginosa*, *Fusobacterium* spp., *Providencia* spp., *Prevotella intermedia*, *Peptostreptococcus magnus*, *E. saphenum*, *Porphyromonas gingivalis*. *Bacteroides* spp., *Clostridium* spp., *Lactobacillus* spp., and *Fusobacterium nucleatum*. We observed that *P. gingivalis* and *P. intermedia* were present only in the rat groups subjected to DSS and fed with an HFD, whereas *Providencia* spp. was absent from these groups compared with the others (Table 1).

### 2.10. Effect of RSVT and LB on the Relative Abundance of Fusobacteria, Bifidobacteria, E. coli, Lactobacillus, Clostridium, and Bacteroides as Determined by qRT-PCR

**Figure 11:**
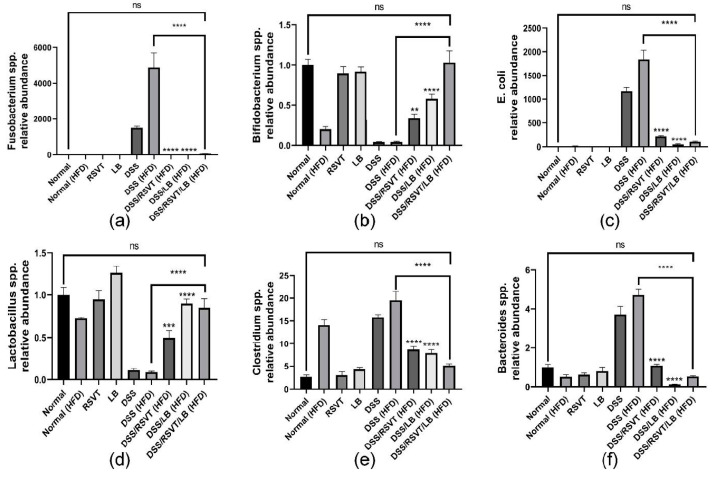
Effect of rosuvastatin (RSVT), *Lactobacillus* (LB), and their combined therapy on the relative abundance of different microbial species (**a**), *Bifidobacterium* spp.; (**b**), *Lactobacillus* spp.; (**c**), *E. coli*; (**d**), *Fusobacterium* spp.; (**e**), *Clostridium* spp.; and (**f**), *Bacteroides* spp. **, *p* < 0.01 vs. DSS (HFD); ***, *p* < 0.001 vs. DSS (HFD); ****, *p* < 0.0001 vs. DSS (HFD); ns, non-significant.

The relative abundance of *Fusobacterium* (Figure 11a)*, Bifidobacterium* (Figure 11b)*, E. coli* (Figure 11c), *Lactobacillus* (Figure 11d), *Clostridium* spp. (Figure 11e), and *Bacteroides* (Figure 11f) were determined. The results indicated a greater abundance of *Fusobacterium* spp., *E. coli*, *Clostridium* spp., and *Bacteroides* spp. in the DSS-treated groups, which were not treated with RSVT, LB, or their combination, whereas *Bifidobacterium* spp. and *Lactobacillus* spp. were markedly abundant in the control samples and treated rat groups.

## 3. Discussion

The innate immune system is the first-line protection that senses microbes or endogenous threat signals through host pattern recognition receptors, such as the TLRs and NOD-like receptors (NLRs), of damage-associated molecular patterns (DAMPs) or pathogen associated molecular patterns (PAMPs). To identify the PAMPs or DAMPs, the NLR family member, NLRP3, plays an important role. Additionally, as a major component of innate immunity, the NLRP3 inflammasome plays a critical role in the inflammatory response, providing a molecular platform that can be triggered by multiple endogenous and exogenous stimuli including ATP, microbial agonists, particulate matter, and pore-forming toxins [22,23]. The NLRP3 inflammasome multiprotein complex consists of the NLRP3 sensor, the speck-like adaptor-apoptosis-associated protein containing a domain of caspase recruitment (ASC), and the effector protein caspase-1. ASC includes a pyrin domain which associates with an upstream region of NLRP3 and a caspase recruitment domain, which associates with caspase-1. Caspase-1 converts the proforms of IL-1β and IL-18 into their corresponding active forms. Moreover, caspase-1 triggers pyroptosis, which results in cellular lysis and the release of cytosolic contents into the extracellular space. TXNIP is a ubiquitously expressed protein that interacts and negatively regulates the expression and function of thioredoxin. Blood and colon tissues from patients with IBD were used to show that TXNIP can distinguish patients with IBD from non-IBD [24]. It is worth noting that TXNIP is an upstream partner to NLRP3 and the association between these two proteins is necessary for downstream inflammasome activation [25]. This inhibits TXNIP effects and provides an additional protective mechanism against UC.

ROS represents a common reaction toward several harmful signals that stimulate TXNIP [26]. Additionally, many studies have indicated that Ox-LDL induces the rapid production of intracellular ROS [27]. Ox-LDL-mediated oxidative stress upregulates TXNIP and subsequently induces binding of TXNIP to NLRP3 to mediate NLRP3 inflammasome assembly and activation [28]. It has been reported that Ox-LDL plasma levels are correlated with colitis severity. Therefore, treatment with statins may represent a strategy to attenuate the inflammatory response in the colon [29]. Additionally, CD36 has been shown to play an essential role by binding and promoting the endocytosis of Ox-LDL [30]. CD36 is a free fatty acid transporter involved in various biological processes including inflammation. Moreover, levels of Ox-LDL are closely associated with increases in the levels of CD36. CD36 ligands (e.g., Ox-LDL) trigger the assembly of a CD36-TLR4-TLR6 heterotrimeric complex, activate NF-κB, and up-regulate NF-κB-mediated NLRP3 expression [31]. Furthermore, CD36-mediated Ox-LDL uptake results in the intracellular accumulation of cholesterol crystals that cause lysosomal disruption and NLRP3 inflammasome activation [32]. NF-κB plays a crucial role in regulating the transcription of multiple genes associated with inflammation [33,34,35,36]. It is one of the dominant players in the process of NLRP3 inflammasome activation and contributes significantly to the pathogenesis of UC. DSS induces the activation of NF-κB through the ROS-IKKβ-mediated pathway and increases the phosphorylation of IκBα to facilitate the nuclear translocation of NF-κB p65 [37]. Additionally, HFD is associated with nuclear retention of NF-κB, which maintains its constitutive activation [38]. HFD increases NLRP3 expression in murine adipose tissue, whereas a calorie-restricted diet decreases its expression [39].

Predisposing factors that promote colon ulceration have been extensively investigated and include dysbiosis of the gut microbiota [40]. Alterations in gut microbiota detected in the form of changes in abundance, diversity, and composition results directly in the reduction of probiotics and an increase in opportunistic pathogens [41]. The microbiota and microbial metabolites are involved in intestinal inflammation [42]. This constitutes a promising strategy for relieving IBDs by controlling dysbiosis. Furthermore, there are promising therapeutic approaches that may help to control UC such as the inflammasome targeting approach. Notably, several studies have reported the detrimental effects of NLRP3 inflammasome dysfunction on microbial homeostasis. Conversely, many reports are suggesting that the microbiota activate NLRP3 and exacerbate colitis [43]. In the present study, the probiotics that were negatively affected by DSS/HFD-induced UC included *Bifidobacterium* spp. and *Lactobacillus* spp. *Bifidobacterium* exerts its effects by maintaining the mucosal barrier that protects the colonic epithelia against inflammation and ulcerative factors [44]. We revealed a significant reduction in the abundance of *Bifidobacterium* in DSS-treated rats. This abundance was restored in the groups subjected to treatment with LB, particularly with RSVT/LB combination therapy. Consistent with our findings, a reduction of *Bifidobacterium* was previously detected when UC was chemically induced [45]. *Lactobacillus* has a significant role in attenuating IBD. Several studies reported a negative correlation between the amount of *Lactobacillus* in active IBD patients which reflects an intrinsic pharmabiotic role to alleviate IBD by correcting homeostasis of the gut microbiota [46]. Our results indicated that RSVT/LB effectively restored the protective environment to maintain the colon in a healthy condition. Conversely, when we investigated the variety of bacterial species that promote dysbiosis, we identified four categories of bacteria (*E. coli*, *Fusobacterium* spp., *Clostridium* spp., and *Bacteroides* spp.) that showed a significant and prompt flourishment in UC induced by DSS exposure. These categories make sense as they corresponding to their pathogenic role and feedback in the manifestation of UC. Adherent and enteropathogenic *E. coli* have also been significantly associated with UC in several studies [47]. In the present study, RSVT/LB reduced the abundance of *E. coli* and prompted a shift to normal levels in healthy mucosa. *Fusobacterium* spp. has also been tightly linked to the clinical severity of UC as it produces secretory proteins that disrupt the integrity of the mucosal barrier. Moreover, they produce butyric acid that irritates and ulcerates the colon [48]. In the present study, the overgrowth of *Fusobacterium* was clearly inhibited upon treatment with RSVT/LB. An important pathogen associated with dysbiosis is *Clostridium* spp., a well-known opportunistic gram-positive anaerobic bacterium that flourishes in an altered microbiome when other gut-beneficial bacteria cannot counterbalance its proliferation. There is a significant association between the incidence and severity of IBD and infection with *Clostridium* spp. The exotoxins secreted by *Clostridium* spp. severely harm the mucosal barrier and expose the epithelial cells to further damage [49]. These findings are suggestive of the microbiome changes, while determination of the *Clostridium* at a species level warrants further investigations. 

An association between dyslipidemia, the oxidation of LDL, CD36, ROS generation, TXNIP upregulation, NLRP3 inflammasome activation, and dysbiosis has been demonstrated in the present study through the action of DSS in HFD-fed rats. Our results demonstrate the aggravation of intestinal inflammation as a consequence of HFD feeding and its relationship to DSS exposure. We investigated for the first time the effect of RSVT and LB alone and in combination for the treatment of DSS/HFD-induced UC. Our results indicated that treatment with RSVT/LB dramatically improved the histological changes and reduced the histological score induced by DSS and HFD. Additionally, RSVT/LB repressed the DSS/HFD-induced increase in colon weight/length ratio, DAI, and MDI. Moreover, RSVT/LB successfully re-established the altered spectrum of the gut microbiome. We believe that RSVT treatment represents a potential strategy to reduce Ox-LDL-induced TXNIP and thus attenuate the inflammatory response by inhibiting NLRP3 inflammasome assembly. Additionally, combining RSVT with LB provides further protection by correcting dysbiosis.

Simultaneous administration of LB with RSVT provides an increased defense mechanism against oxidative stress and decreases myeloperoxidase activity. Additionally, the NLRP3 inflammasome effector protein, caspase-1, expression, and activity were effectively subdued upon treatment with combined RSVT/LB therapy. Consequently, IL-1β-driven pyroptotic activity was ameliorated. Moreover, combined RSVT/LB therapy showed a prominent anti-inflammatory potential as revealed by the IL-10/IL-12 P70 ratio and the level of TNF-α and IL-6. The latter effects may be attributed to the inhibiting of phosphorylation-induced activation of NF-κB p65 resulting in a reduction in the expression of NLRP3 and the proforms of IL-1β and IL-18. To conclude, combination therapy using Lactobacillus as an adjunctive to rosuvastatin offers a safe and effective therapeutic strategy for the management of UC, particularly in colitic patients with dyslipidemia. Therefore, this novel approach warrants further investigation in a clinical setting.

## 4. Materials and Methods

### 4.1. Experimental Study and Treatment Protocol

#### 4.1.1. Animals

Protocols for the treatment and use of laboratory animals were approved by the Research Ethics Committee at the Faculty of Pharmacy, Delta University for Science and Technology, Egypt (approval number, FPDU24120) which followed ARRIVE guidelines, Animal Research Reporting of In Vivo Experiments, and carried out in accordance with the U.K. animals (scientific procedures) Act 1986 and the associated guidelines of EU directive 2010/63/EU for animal experiments. The study utilized adult male Sprague-Dawley rats obtained from the Theodor Bilhars Research Institute, Giza, Egypt. Rats were weighed (260 ± 20 g) and held in polypropylene cages for 6 weeks under identical environmental conditions and allowed free access to normal diet or HFD and water ad libitum. 

#### 4.1.2. HFD Composition

As described by Keshk, et al. [50], the HFD comprised 20% protein, 35% carbohydrates (18% sucrose, 10% starch,, and 7% maltodextrin), and 45% lipids based on dry weight.

#### 4.1.3. Preparation of Lactobacillus Suspension

A sachet containing *Lactobacillus fermentum* and *Lactobacillus delbrueckii* (1 × 10^10^ microbial cells) was dissolved in pathogen-free drinking water to prepare a probiotic suspension [51]. Each rat received 0.5 mL of the microbial suspension containing 2.7 × 10^8^ CFU/mL, which was administered to the rats p.o. [52].

#### 4.1.4. Experimental Design

As shown in Table 2, following the acclimatization phase, rats were divided into nine groups: Normal group, in which rats were allowed free access to a normal diet (ND) for 16 days (*n* = 6); Normal (HFD) group, in which rats were allowed free access to an HFD for 16 days (*n* = 6); RSVT group, in which animals were administered RSVT (20 mg/kg/day, p.o. as a calcium salt, Sigma-Aldrich, St. Louis, MO, USA) and allowed free access to an ND for 16 days (*n* = 6); LB group, in which rats were administered Lactobacillus (0.5 mL/day, p.o.) (Rameda Pharma Co., Giza, Egypt) and allowed free access to an ND for 16 days (*n* = 6); DSS group, in which rats were allowed free access to an ND for 16 days and free access to drinking water containing 4% DSS (*w*/*v*) (Sigma-Aldrich, St. Louis, MO, USA) for 7 days starting from day 3 to day 9 (*n* = 10); DSS (HFD) group, in which rats were allowed free access to an HFD for 16 days and free access to drinking water containing 4% DSS for 7 days starting from day 3 to day 9 (*n* = 10); DSS/RSVT (HFD) group, in which animals were administered RSVT (20 mg/kg/day, p.o.) and were allowed free access to an HFD for 16 days and free access to drinking water containing 4% DSS for 7 days starting from day 3 to day 9 (*n* = 8); DSS/LB (HFD) group, in which rats were administered LB (0.5 mL/day, p.o.) and were allowed free access to an HFD for 16 days and free access to drinking water containing 4% DSS for 7 days starting from day 3 to day 9 (*n* = 8); and DSS/RSVT/LB (HFD) group, in which animals were administered RSVT (20 mg/kg/day, p.o.) + LB (0.5 mL/day, p.o.) and were allowed free access to HFD for 16 days and free access to drinking water containing 4% DSS for 7 days starting from day 3 to day 9 (*n* = 8).

#### 4.1.5. Sample Collection and Preparation

At the end of experiment, colons were dissected and weighed, and the lengths were measured. After blood collection, sera were isolated and stored at −80 °C for biochemical experiments. Using ice-cold saline, the fresh colons were cleaned and dried on clean paper towels. The colons were divided into two portions (distal colon): one portion was preserved in 4% neutral-buffered formalin for histopathological and immunohistochemical investigations, whereas the second portion was immediately frozen in liquid nitrogen and stored at −80 °C for qRT-PCR, ELISA, and colorimetric assays. Additionally, 300 mg cecum stool samples were collected from each rat immediately after colon dissection. DNA was extracted using the QIAamp DNA Stool Mini Kit (cat. # 51504, Qiagen Inc., Hilden, Germany) following the manufacturer’s guidelines. DNA extracts were spectrophotometrically analyzed with a NanoDrop instrument (OPTIZEN NanoQ, Mecasys Co., Ltd, Daejeon, Korea) to measure DNA concentration.

### 4.2. Assessment of Disease Activity Index and Macroscopic Damage Index

The disease activity index (DAI) was used for the assessment of disease intensity, which quantified a percent body weight loss score relative to that measured just before the induction of colitis, stool consistency, and gross bleeding. Each parameter was scored as follows: diarrhea (0, normal; 1 and 2, loose stools; and 3 and 4, watery diarrhea); percentage body weight loss (0, none; 1, 1–5%; 2, 6–10%; 3, 11–20%; and 4, >20%); bloody stool (0, normal; 1 and 2, slight bleeding; and 3 and 4, gross bleeding) [53]. Macroscopic damage index (MDI) was assessed as described previously by Saber and El-Kader [26]. In brief, MDI is calculated as the sum of each animal score. This scoring system was based on a single-blinded visual evaluation of the intestinal damage. The MDI scoring criteria for the colonic macroscopic damage was based on an arbitrary scale ranging from 0 to 4. The scoring criteria was as follows: No macroscopic features, 0; Presence of mucosal erythema only, 1; Presence of mild mucosal edema and slight mucosal bleeding or erosions, 2; Presence of moderate mucosal edema with moderate mucosal bleeding or erosions, 3; Presence of severe edema and tissue necrosis, 4.

### 4.3. Histological Examination and Immunohistochemical Labeling of NF-кB p65 and Caspase-1

Standard histological procedures were followed for the preparation and staining of colon tissue specimens with hematoxylin and eosin and a scoring system was established for the assessment of the microscopic intestinal damage [54]. The adopted scoring criteria were as follows: No histopathological signs of inflammation, 0; Mild inflammation, 1; Mild leucocytes infiltration, 2; Severe leucocytes infiltration, high vascularity, and increased colonic thickness, 3; loss of goblet cells, severe leucocytes infiltration, high vascularity, and increased colonic thickness, 4. Additionally, guidelines provided by Saber et al. [27] were followed for the immunohistochemical labeling of NF-кB p65 and caspase-1 using caspase-1 (R&D Systems Inc., Minneapolis, MN, USA; 1:100 dilution) and NFκB p65 (Rel A, ab-1 rabbit polyclonal Thermo Fisher Scientific, Waltham, MA, USA, at dilution 1:100) primary antibodies. The labeling index of NF-κB p65 and caspase-1 were presented as the % of positive cells/total 1000 counted cells in 10 HPFs.

### 4.4. Biochemical Analysis

#### 4.4.1. Assessment of Colonic Myeloperoxidase Activity

A phosphate-buffered saline, pH 5.4 was used for the preparation of a colon tissue homogenate. In the activity assay kit provided by Sigma-Aldrich, MPO catalyzes the formation of hypochlorous acid, which reacts with taurine to form taurine chloroamine. Taurine chloroamine reacts with the chromophore TNB, resulting in the formation of the colorless product DTNB. One unit of MPO activity is defined as the amount of enzyme that hydrolyzes the substrate and generates taurine chloramine to consume 1.0 μmole of TNB per minute at 25 °C.

#### 4.4.2. Assessment of Oxidative Stress Markers and Lipid Profile

Superoxide dismutase (SOD), reduced glutathione (GSH), and malondialdehyde (MDA) were assayed in the homogenate with kits purchased from Bio-diagnostic (Giza, Egypt). The assays were performed following the manufacturer’s guidelines. Reactive oxygen species (ROS) in colon tissues was assessed as previously described [55]. Briefly, colon samples (200 mg) were homogenized in ice-cold Tris-HCl buffer (40 mM, pH = 7.4) (1:10 *w*/*v*). Then, homogenates (100 µL) were mixed with Tris-HCl buffer (1 mL), and 5 µL of 2′, 7′-dichlorofluorescein diacetate (10 µM) (Sigma-Aldrich) was added and the reactions were incubated for 30 min at 37 °C. The fluorescence intensity (FI) was measured with a SpectraFluor Plus Microplate Reader (Tecan, Mainz, Germany), λ excitation = 485 nm and λ emission = 525 nm). Total cholesterol, triglycerides, and high-density lipoprotein (HDL) cholesterol were measured in sera using kits purchased from Bio-diagnostic following the manufacturer’s recommendations.

#### 4.4.3. Assessment of Caspase-1 Activity

A kit was obtained from R&D Systems (Minneapolis, MN, USA) to evaluate caspase-1 activity based on the detection of cleaved p-nitroanilide (p-NA) chromophore following the manufacturer’s instructions.

#### 4.4.4. Assessment of IL-1β, IL-6, IL-18, TNF-α, IL-10, IL-12 p70, TXNIP, CD36, and Ox-LDL by ELISA

IL-1β was assayed using an ELISA kit purchased from BioLegend (San Diego, CA, USA). IL-6, IL-18, TNF-α, and IL-10 were measured using kits obtained from eBioscience (Vienna, Austria). IL-12 p70, thioredoxin-interacting protein (TXNIP), and circulating and colon associated CD36 were assayed using ELISA kits from CUSABIO (Wuhan, China) following the manufacturer’s instructions. Levels of serum Ox-LDL were measured using a kit obtained from Elabscience (Wuhan, China) following the recommended protocol.

#### 4.4.5. Quantitative Real-Time PCR for the Expression of NLPR3, TXNIP, and NF-кB P65 in Colon Tissue

Total RNA was extracted from colon tissues using an RNeasy Mini kit (Qiagen) in an RNase-free environment according to the manufacturer’s instructions. The RNA quantity and purity were assessed using a NanoDrop 2000 spectrophotometer (Thermo Fisher Scientific, Wilmmington, DE, USA). RNA (1 μg) was reverse-transcribed using Quantiscript reverse transcriptase (QuantiTect Reverse Transcription Kit, Qiagen). The PCR reactions were done with a Rotor-Gene Q thermocycler (Qiagen) using SYBR Green PCR mastermMix (Qiagen). The thermal cycling conditions were as follows: 3 min at 95 °C, 40 cycles at 95 °C for 10 s, the annealing temperatures listed in Table 3, and a 72 °C step for 30 s. Relative expression was calculated using the comparative cycle threshold (C_t_) (2^−ΔΔCT^) method. All values were normalized to GAPDH expression as an invariant endogenous control. The Rotor-Gene Q Software 2.1 (Qiagen) was used for data analysis, and Table 3 provides the sequences of the primer pairs.

#### 4.4.6. Conventional PCR for the Detection of Gut Microbiota

Reaction mixtures (25 μL) containing DNA samples were prepared for thermal cycling according to the following protocol: 12.5 µL my Taq red mix (Bioline Co., London, UK), 1 μL of each primer (10 μM each), 2.5 µL DNA, and nuclease-free water. The PCR amplification protocol was as follows: initial denaturation step at 94 °C for 5 min, 35 cycles at 94 °C for 30 s, annealing temperature calculated for each primer mix as indicated in Table 4 for 30 s, extension at 72 °C for 45 s, and a final termination step at 72 °C for 3 min. The expected PCR amplicons were detected by electrophoretic separation on 1.5% agarose gel and compared with a GeneRuler 100 bp plus DNA ladder (Thermo Fisher Scientific) [56]. The gels were stained with ethidium bromide and visualized with a UV transilluminator. Table 4 lists the primer sequences specific for the various bacteria.

#### 4.4.7. Quantitative Real-Time PCR for the Detection of the Relative Abundance of *Fusobacteria, Bifidobacteria*, *E. coli*, *Lactobacillus, Clostridium*, and Bacteroides

Quantitative assessment of specific gut bacteria species was done using primers for the 16S rDNA housekeeping gene as a proxy for bacterial abundance. Extracted fecal DNA (40–80 ng) was mixed with 12.5 μL (2×) SYBR Green PCR master mix (Willowfort Co., Birmingham, UK), 1.5 μL of forward and reverse primer (10 μmol each), and 7.5 μL nuclease-free H_2_O to a final volume of 25 μL. All reactions were performed using a MyGo real-time PCR machine with the following cycling protocol: 95 °C for 5 min, followed by 45 cycles of 95 °C for 20 s, annealing for 20 s, and 72 °C for 40 s. The Ct values and melting curves were obtained using the MyGo real-time PCR machine software. The relative abundance of each microbial species was calculated as a relative unit normalized to the total bacteria of the corresponding sample using the 2^−ΔΔCt^ method (where ΔCt = the average Ct value of each target—the average Ct value of total bacteria) [56]. Table 4 lists the primer sequences for the detection of the different bacterial strains.

### 4.5. Statistical Analysis 

Statistical analysis was performed using GraphPad Prism software version 6 (GraphPad Software Inc., La Jolla, CA, USA). Differences between groups were analyzed by one-way analysis of variance (ANOVA) followed by Tukey’s Kramer multiple comparison test. Data are presented as mean ± standard deviation (SD). Kruskal-Wallis test followed by Dunn’s post-hoc test was performed to analyze differences between groups for the histological score of inflammation, disease activity and macroscopic damage indices. Data are presented as median (IQR). All the analytical measurements were performed using samples collected from 6 rats of an experimental group. Spectrophotometric and ELISA measurements were run once and either the conventional or real time PCR measurements were performed in duplicates. A value of *p* ≤ 0.05 was considered to indicate statistical significance.

## 5. Conclusions

Combination therapy using Lactobacillus as an adjunctive to rosuvastatin might serve as a safe and effective therapeutic strategy for the management of Ulcerative colitis.

## Figures and Tables

**Table 1:** Alterations in the gut microbiome composition as detected by conventional PCR.

Organism	Normal	Normal (HFD)	RSVT	LB	DSS	DSS (HFD)	DSS/RSVT (HFD)	DSS/LB (HFD)	DSS/RSVT/LB (HFD)
*Enterococcus faecalis*	**−**	**−**	**−**	**−**	**−**	**−**	**−**	**−**	**−**
*Enterococcus faecium*	**−**	**−**	**−**	**−**	**−**	**−**	**−**	**−**	**−**
*Escherichia coli*	**+**	**+**	**+**	**+**	**+**	**+**	**+**	**+**	**+**
*Bifidobacterium* spp.	**+**	**+**	**+**	**+**	**+**	**+**	**+**	**+**	**+**
*Pseudomonas* *aeruginosa*	**−**	**+**	**−**	**−**	**+**	**+**	**−**	**−**	**−**
*Fusobacterium* spp.	**+**	**+**	**+**	**+**	**+**	**+**	**+**	**+**	**+**
*Providencia* spp.	**+**	**−**	**+**	**+**	**−**	**−**	**+**	**+**	**+**
*Prevotella intermedia*	**−**	**+**	**−**	**−**	**+**	**+**	**−**	**−**	**−**
*Peptostreptococcus magnus*	**−**	**−**	**−**	**−**	**−**	**−**	**−**	**−**	**−**
*Eubacterium saphenum*	**−**	**−**	**−**	**−**	**−**	**−**	**−**	**−**	**−**
*Porphyromonas gingivalis*	**−**	**+**	**−**	**−**	**+**	**+**	**−**	**−**	**−**
*Bacteroides* spp.	**+**	**+**	**+**	**+**	**+**	**+**	**+**	**+**	**+**
*Clostridium* spp.	**+**	**+**	**+**	**+**	**+**	**+**	**+**	**+**	**+**
*Lactobacillus* spp.	**+**	**+**	**+**	**+**	**+**	**+**	**+**	**+**	**+**
*Fusobacterium nucleatum*	**−**	**−**	**−**	**−**	**−**	**−**	**−**	**−**	**−**

**+**, detected; **−**, not detected; DSS, dextran sodium sulphate; HFD, high fat diet; LB, lactobacillus; ND, normal diet; RSVT, rosuvastatin.

**Table 2:** Experimental design and treatment protocol.

Exp. Groups	Day 1–Day 2	Day 3–Day 9	Day 10–Day 16
Normal (*n* = 6)	ND	ND	ND
Normal (HFD) (*n* = 6)	HFD	HFD	HFD
RSVT (*n* = 6)	RSVT (20 mg/kg/day, p.o.) + ND	RSVT (20 mg/kg/day, p.o.) + ND	RSVT (20 mg/kg/day, p.o.) + ND
LB (*n* = 6)	LB (0.5 mL, p.o.) + ND	LB (0.5 mL, p.o.) + ND	LB (0.5 mL, p.o.) + ND
DSS (*n* = 10)	ND	4% DSS in drinking water + ND	ND
DSS (HFD) (*n* = 10)	HFD	4% DSS in drinking water + HFD	HFD
DSS/RSVT (HFD) (*n* = 8)	RSVT (20 mg/kg/day, p.o.) + HFD	RSVT (20 mg/kg/day, p.o.) + 4% DSS in drinking water + HFD	RSVT (20 mg/kg/day, p.o.) + HFD
DSS/LB (HFD) (*n* = 8)	LB (0.5 mL, p.o.) + HFD	LB (0.5 mL, p.o.) + 4% DSS in drinking water + HFD	LB (0.5 mL, p.o.) + HFD
DSS/RSVT/LB (HFD) (*n* = 8)	RSVT (20 mg/kg/day, p.o.) + LB (0.5 mL, p.o.) + HFD	RSVT (20 mg/kg/day, p.o.) + LB (0.5 mL, p.o.) + 4% DSS in drinking water + HFD	RSVT (20 mg/kg/day, p.o.) + LB (0.5 mL, p.o.) + HFD

DSS, dextran sodium sulphate; HFD, high-fat diet; LB, lactobacillus; ND, normal diet; RSVT, rosuvastatin.

**Table 3:** Primer sequences for qRT-PCR.

Primer	GenBank Accession	F	R	Ta (°C)	Amplicon Size (bp)
TXNIP	NM_001008767.1	5′-AAGCTGTCCTCAGTCAGAGGCAAT-3′	5′-ATGACTTTCTTGGAGCCAGGGACA-3′	64	170
NLRP3	NM_001191642.1	5′-GAGCTGGACCTCAGTGACAATGC-3′	5′-ACCAATGCGAGATCCTGACAACAC-3′	63	146
NFκB p65	NM_199267.2	5′-TTCCCTGAAGTGGAGCTAGGA-3′	5′-CATGTCGAGGAAGACACTGGA-3′	61	185
GAPDH	NM_017008.4	5′-TCAAGAAGGTGGTGAAGCAG-3′	5′-AGGTGGAAGAATGGGAGTTG-3′	57	111

**Table 4:** Primer sequences for the detection of different species of bacteria.

Primer Name	Forward and Reverse (5’ to 3’)	Primer Sequence	Ta (°C)	Ampilicon Size (bp)
(16S)	F	GAGTTTGATCCTGGCTCAG	51	312
	R	GCTGCCTCCCGTAGGAGT		
*Porphyromonas gingivalis*	F	AATCGTAACGGGCGACACAC	53	594
	R	GGGTTGCTCCTTCATCACAC		
*E. saphenum*	F	AACCACATAAAATCATAGG	43	828
	R	ATACCCGATTAAGGGTAC		
*Fusobacterium*	F	GGATTTATTGGGCGTAAAGC	51.5	162
	R	GGCATTCCTACAAATATCTACGAA		
*E. coli*	F	TGGGAGCGAAAATCCTG	47.5	219
	R	CAGTACAGGTAGACTTCTG		
*Providencia*	F	ACCGCATAATCTCTTAGG	43.5	514
	R	CTACACATGGAATTCTAC		
*E. Faecium*	F	GCAAGGCTTCTTAGAGA	46.5	564
	R	CATCGTGTAAGCTAACTTC		
*Bifidobacterium*	F	CTCCTGGAAACGGGTGG	51	551
	R	GGTGTTCTTCCCGATATCTACA		
*Prevotella intermedia*	F	CGAACCGTCAAGCATAGGC	54	368
	R	AACAGCCGCTTTTAGAACACAA		
*Peptostreptococcus magnus*	F	CGGGNTTTAGTAGACAGAAG	50	565
	R	CAGTTTCCAATGCTTTACGG		
*P.aeruginosa*	F	CGAGTACAACATGGCTCTGG	53	116
	R	ACCGGACGCTCTTTACCATA		
*E. faecalis*	F	ATCAAGTACAGTTAGTCTT	44	940
	R	ACGATTCAAAGCTAACTG		
*Bacteroides* spp.	F	AAGGGAGCGTAGATGGATGTTTA	55	193
	R	CGAGCCTCAATGTCAGTTGC		
*Clostridium* spp.	F	CGGTACCTGACTAAGAAGC	50	429
	R	AGTTTGATTCTTGCGAACG		
*Lactobacillus* spp.	F	AGCAGTAGGGAATCTTCCA	50	334
	R	CACCGCTACACATGGAG		
*Fusobacterium nucleatum*	F	CAACCATTACTTTAACTCTACCATGTTCA	57	356
	R	GTTGACTTTACAGAAGGAGATTATGTAAAAATC		

## Data Availability

The data presented in this study are available on request from the corresponding author.

## Abbreviations

CARD, caspase recruitment domain; CD36, cluster of differentiation 36; CD, Chron’s disease; CFU, Colony forming unit; DAI, Disease activity index; DAMPs, damage-associated molecular patterns; DSS, Dextran sodium sulfate; GSH, reduced glutathione; HFD, High-fat diet; HMG CoA, 3-hydroxy-3-methylglutaryl coenzyme A; IBD, Inflammatory Bowel disease; IL, Interleukin; LAB, Lactic acid bacteria; LB, Lactobacillus; LDL, Low density lipoprotein; MDA, malondialdehyde; MPO, Myeloperoxidase; NF-κB, Nuclear factor kappa B; NLRP3, NOD-, LRR- and pyrin domain-containing protein 3; PAMPs, pathogen-associated molecular patterns; ROS, Reactive oxygen species; PRRs, pattern recognition receptors; RSVT, Rosuvastatin; SCFA, Short chain fatty acids; SOD, Superoxide dismutase; TLRs, Toll-like receptors; TNF-α, Tumor necrosis factor alpha; TXNIP, Thioredoxin interacting protein; UC, Ulcerative colitis.

## References

[1] Saber S., Khalil R.M., Abdo W.S., Nassif D., El-Ahwany E. (2019). Olmesartan ameliorates chemically-induced ulcerative colitis in rats via modulating NFκB and Nrf-2/HO-1 signaling crosstalk. Toxicol. Appl. Pharmacol..

[2] Saber S., Youssef M.E., Sharaf H., Amin N.A., El-Shedody R., Aboutouk F.H., El-Galeel Y.A., El-Hefnawy A., Shabaka D., Khalifa A. (2021). BBG enhances OLT1177-induced NLRP3 inflammasome inactivation by targeting P2X7R/NLRP3 and MyD88/NF-κB signaling in DSS-induced colitis in rats. Life Sci..

[3] Kayal M., Shah S. (2019). Ulcerative Colitis: Current and Emerging Treatment Strategies. J. Clin. Med..

[4] Sartor R.B. (2006). Mechanisms of Disease: Pathogenesis of Crohn’s disease and ulcerative colitis. Nat. Clin. Pract. Gastroenterol. Hepatol..

[5] Tourkochristou E., Aggeletopoulou I., Konstantakis C., Triantos C. (2019). Role of NLRP3 inflammasome in inflammatory bowel diseases. World J. Gastroenterol..

[6] Zhen Y., Zhang H. (2019). NLRP3 Inflammasome and Inflammatory Bowel Disease. Front. Immunol..

[7] Lewis J.D., Abreu M.T. (2017). Diet as a Trigger or Therapy for Inflammatory Bowel Diseases. Gastroenterology.

[8] Chatauret N., Favreau F., Giraud S., Thierry A., Rossard L., Le Pape S., Lerman L.O., Hauet T. (2014). Diet-induced increase in plasma oxidized LDL promotes early fibrosis in a renal porcine auto-transplantation model. J. Transl. Med..

[9] Kashyap P.C., Reigstad C.S., Loftus E.V. (2013). Role of diet and gut microbiota in management of inflammatory bowel disease in an Asian migrant. J. Allergy Clin. Immunol..

[10] Albenberg L.G., Lewis J.D., Wu G.D. (2012). Food and the gut microbiota in inflammatory bowel diseases. Curr. Opin. Gastroenterol..

[11] Gulhane M., Murray L., Lourie R., Tong H., Sheng Y.H., Wang R., Kang A., Schreiber V., Wong K.Y., Magor G. (2016). High Fat Diets Induce Colonic Epithelial Cell Stress and Inflammation that is Reversed by IL-22. Sci. Rep..

[12] Guo X.Y., Liu X.J., Hao J.Y. (2020). Gut microbiota in ulcerative colitis: Insights on pathogenesis and treatment. J. Dig. Dis..

[13] Saber S. (2018). Angiotensin II: A key mediator in the development of liver fibrosis and cancer. Bull. Natl. Res. Cent..

[14] Saber S., Ghanim A.M.H., El-Ahwany E., El-Kader E.M.A. (2020). Novel complementary antitumour effects of celastrol and metformin by targeting IκBκB, apoptosis and NLRP3 inflammasome activation in diethylnitrosamine-induced murine hepatocarcinogenesis. Cancer Chemother. Pharmacol..

[15] Younis N.S., Ghanim A.M.H., Saber S. (2019). Mebendazole augments sensitivity to sorafenib by targeting MAPK and BCL-2 signalling in n-nitrosodiethylamine-induced murine hepatocellular carcinoma. Sci. Rep..

[16] Ni J., Wu G.D., Albenberg L., Tomov V.T. (2017). Gut microbiota and IBD: Causation or correlation?. Nat. Rev. Gastroenterol. Hepatol..

[17] Azad M.A.K., Sarker M., Li T., Yin J. (2018). Probiotic Species in the Modulation of Gut Microbiota: An Overview. BioMed Res. Int..

[18] Hansen J.J., Sartor R.B. (2015). Therapeutic Manipulation of the Microbiome in IBD: Current Results and Future Approaches. Curr. Treat. Options Gastroenterol..

[19] Shin S.K., Cho J.H., Kim E.J., Kim E.-K., Park D.K., Kwon K.A., Chung J.-W., Kim K.O., Kim Y.J. (2017). Anti-inflammatory and anti-apoptotic effects of rosuvastatin by regulation of oxidative stress in a dextran sulfate sodium-induced colitis model. World J. Gastroenterol..

[20] Chen X., Zhao X., Wang H., Yang Z., Li J., Suo H. (2017). Prevent Effects of Lactobacillus Fermentum HY01 on Dextran Sulfate Sodium-Induced Colitis in Mice. Nutrients.

[21] Ungaro R., Chang H.L., Cote-Daigneaut J., Mehandru S., Atreja A., Colombel J.-F. (2016). Statins Associated with Decreased Risk of New Onset Inflammatory Bowel Disease. Am. J. Gastroenterol..

[22] Zhang Y., Tan L., Li C., Wu H., Ran D., Zhang Z. (2020). Sulforaphane alter the microbiota and mitigate colitis severity on mice ulcerative colitis induced by DSS. AMB Express.

[23] El-Baz A.M., Khodir A.E., El-Sokkary M.M.A., Shata A. (2020). The protective effect of Lactobacillus versus 5-aminosalicylic acid in ulcerative colitis model by modulation of gut microbiota and Nrf2/Ho-1 pathway. Life Sci..

[24] Requena T., Martinez-Cuesta M.C., Peláez C. (2018). Diet and microbiota linked in health and disease. Food Funct..

[25] Wagatsuma K., Nakase H. (2020). Contradictory Effects of NLRP3 Inflammasome Regulatory Mechanisms in Colitis. Int. J. Mol. Sci..

[26] Saber S., El-Kader E.M.A. (2020). Novel complementary coloprotective effects of metformin and MCC950 by modulating HSP90/NLRP3 interaction and inducing autophagy in rats. Inflammopharmacology.

[27] Saber S., El-Kader E.M.A., Sharaf H., El-Shamy R., El-Saeed B., Mostafa A., Ezzat D., Shata A. (2020). Celastrol augments sensitivity of NLRP3 to CP-456773 by modulating HSP-90 and inducing autophagy in dextran sodium sulphate-induced colitis in rats. Toxicol. Appl. Pharmacol..

[28] Palmer N.P., Silvester J.A., Lee J.J., Beam A.L., Fried I., Valtchinov V.I., Rahimov F., Kong S.W., Ghodoussipour S., Hood H.C. (2019). Concordance between gene expression in peripheral whole blood and colonic tissue in children with inflammatory bowel disease. PLoS ONE.

[29] Zhou R., Tardivel A., Thorens B., Choi I., Tschopp J. (2010). Thioredoxin-interacting protein links oxidative stress to inflammasome activation. Nat. Immunol..

[30] Nasoohi S., Ismael S., Ishrat T. (2018). Thioredoxin-Interacting Protein (TXNIP) in Cerebrovascular and Neurodegenerative Diseases: Regulation and Implication. Mol. Neurobiol..

[31] Lara-Guzmán O.J., Gil-Izquierdo Á., Medina S., Osorio E., Álvarez-Quintero R., Zuluaga N., Oger C., Galano J.-M., Durand T., Muñoz-Durango K. (2018). Oxidized LDL triggers changes in oxidative stress and inflammatory biomarkers in human macrophages. Redox Biol..

[32] Nyandwi J.B., Ko Y.S., Jin H., Yun S.P., Park S.W., Kim H.J. (2020). Rosmarinic acid inhibits oxLDL-induced inflammasome activation under high-glucose conditions through downregulating the p38-FOXO1-TXNIP pathway. Biochem. Pharmacol..

[33] Cherfane C.E., Gessel L., Cirillo D., Zimmerman M.B., Polyak S. (2015). Monocytosis and a Low Lymphocyte to Monocyte Ratio Are Effective Biomarkers of Ulcerative Colitis Disease Activity. Inflamm. Bowel Dis..

[34] Ramos-Arellano L.E., Muñoz-Valle J.F., De La Cruz-Mosso U., Salgado-Bernabé A.B., Castro-Alarcón N., Parra-Rojas I. (2014). Circulating CD36 and oxLDL levels are associated with cardiovascular risk factors in young subjects. BMC Cardiovasc. Disord..

[35] Zhao L., Varghese Z., Moorhead J.F., Chen Y., Ruan X.Z. (2018). CD36 and lipid metabolism in the evolution of atherosclerosis. Br. Med. Bull..

[36] Sheedy F.J., Grebe A., Rayner K.J., Kalantari P., Ramkhelawon B., Carpenter S.B., Becker C.E., Ediriweera H.N., Mullick A.E., Golenbock D.T. (2013). CD36 coordinates NLRP3 inflammasome activation by facilitating intracellular nucleation of soluble ligands into particulate ligands in sterile inflammation. Nat. Immunol..

[37] El-Gizawy S.A., Nouh A., Saber S., Kira A.Y. (2020). Deferoxamine-loaded transfersomes accelerates healing of pressure ulcers in streptozotocin-induced diabetic rats. J. Drug Deliv. Sci. Technol..

[38] Khalil R., Shata A., El-Kader E.M.A., Sharaf H., Abdo W.S., Amin N.A., Saber S. (2020). Vildagliptin, a DPP-4 inhibitor, attenuates carbon tetrachloride-induced liver fibrosis by targeting ERK1/2, p38α, and NF-κB signaling. Toxicol. Appl. Pharmacol..

[39] Saber S., Goda R., El-Tanbouly G.S., Ezzat D. (2018). Lisinopril inhibits nuclear transcription factor kappa B and augments sensitivity to silymarin in experimental liver fibrosis. Int. Immunopharmacol..

[40] Saber S., Mahmoud A., Helal N., El-Ahwany E., Abdelghany R. (2018). Liver Protective Effects of Renin-Angiotensin System Inhibition Have No Survival Benefits in Hepatocellular Carcinoma Induced by Repetitive Administration of Diethylnitrosamine in Mice. Open Access Maced. J. Med. Sci..

[41] Bhattacharyya S., Dudeja P.K., Tobacman J.K. (2009). ROS, Hsp27, and IKKβ mediate dextran sodium sulfate (DSS) activation of IκBa, NFκB, and IL-8. Inflamm. Bowel Dis..

[42] Shankar E., Vykhovanets E.V., Vykhovanets O.V., MacLennan G.T., Singh R., Bhaskaran N., Shukla S., Gupta S. (2012). High-fat diet activates pro-inflammatory response in the prostate through association of Stat-3 and NF-κB. Prostate.

[43] Rheinheimer J., de Souza B.M., Cardoso N.S., Bauer A.C., Crispim D. (2017). Current role of the NLRP3 inflammasome on obesity and insulin resistance: A systematic review. Metab. Clin. Exp..

[44] Shen Z.-H., Zhu C.-X., Quan Y.-S., Yang Z.-Y., Wu S., Luo W.-W., Tan B., Wang X.-Y. (2018). Relationship between intestinal microbiota and ulcerative colitis: Mechanisms and clinical application of probiotics and fecal microbiota transplantation. World J. Gastroenterol..

[45] Schierová D., Březina J., Mrázek J., Fliegerová K.O., Kvasnová S., Bajer L., Drastich P.J.C. (2020). Gut Microbiome Changes in Patients with Active Left-Sided Ulcerative Colitis after Fecal Microbiome Transplantation and Topical 5-aminosalicylic Acid Therapy. Cells.

[46] Leccese G., Bibi A., Mazza S., Facciotti F., Caprioli F., Landini P., Paroni M.J.C. (2020). Probiotic Lactobacillus and Bifidobacterium Strains Counteract Adherent-Invasive Escherichia coli (AIEC) Virulence and Hamper IL-23/Th17 Axis in Ulcerative Colitis, but Not in Crohn’s Disease. Cells.

[47] Jensen S.R., Mirsepasi-Lauridsen H.C., Thysen A.H., Brynskov J., Krogfelt K.A., Petersen A.M., Pedersen A.E., Brix S. (2015). Distinct inflammatory and cytopathic characteristics of Escherichia coli isolates from inflammatory bowel disease patients. Int. J. Med. Microbiol..

[48] Ohkusa T., Sato N., Ogihara T., Morita K., Ogawa M., Okayasu I. (2002). Fusobacterium varium localized in the colonic mucosa of patients with ulcerative colitis stimulates species-specific antibody. J. Gastroenterol. Hepatol..

[49] Singh H., Nugent Z., Yu B.N., Lix L.M., Targownik L.E., Bernstein C.N. (2017). Higher Incidence of Clostridium difficile Infection Among Individuals With Inflammatory Bowel Disease. Gastroenterology.

[50] Keshk W.A., Ibrahim M.A., Shalaby S.M., Zalat Z.A., Elseady W.S. (2020). Redox status, inflammation, necroptosis and inflammasome as indispensable contributors to high fat diet (HFD)-induced neurodegeneration; Effect of N-acetylcysteine (NAC). Arch. Biochem. Biophys..

[51] Garcia-Castillo V., Komatsu R., Clua P., Indo Y., Takagi M., Salva S., Islam A., Alvarez S., Takahashi H., Garcia-Cancino A. (2019). Evaluation of the Immunomodulatory Activities of the Probiotic Strain Lactobacillus fermentum UCO-979C. Front. Immunol..

[52] Fooladi A.A.I., Yazdi M.H., Pourmand M.R., Mirshafiey A., Hassan Z.M., Azizi T., Mahdavi M., Dallal M.M.S. (2015). Th1 Cytokine Production Induced by Lactobacillus acidophilus in BALB/c Mice Bearing Transplanted Breast Tumor. Jundishapur J. Microbiol..

[53] Cooper H.S., Murthy S.N., Shah R.S., Sedergran D.J. (1993). Clinicopathologic study of dextran sulfate sodium experimental murine colitis. Lab. Investig..

[54] Saber S., Basuony M., Eldin A.S. (2019). Telmisartan ameliorates dextran sodium sulfate-induced colitis in rats by modulating NF-κB signalling in the context of PPARγ agonistic activity. Arch. Biochem. Biophys..

[55] Heidari R., Taheri V., Rahimi H.R., Yeganeh B.S., Niknahad H., Najibi A. (2015). Sulfasalazine-induced renal injury in rats and the protective role of thiol-reductants. Ren. Fail..

[56] Wong S.H., Kwong T.N.Y., Chow T.-C., Luk A.K.C., Dai R.Z.W., Nakatsu G., Lam T.Y.T., Zhang L., Wu J.C.Y., Chan F.K.L. (2016). Quantitation of faecalFusobacteriumimproves faecal immunochemical test in detecting advanced colorectal neoplasia. Gut.

